# Research on a dynamic early warning model based on refined threshold analysis: Case study of the Tanjiawan Landslide

**DOI:** 10.1371/journal.pone.0339689

**Published:** 2026-02-09

**Authors:** Wenjian Wang, Wu Yi, Xiaohu Huang, Yating Wang, Zhengyu Wang

**Affiliations:** 1 Key Laboratory of Geological Hazards on Three Gorges Reservoir Area (China Three Gorges University), Ministry of Education, Yichang, Hubei, China; 2 College of Civil Engineering and Architecture, China Three Gorges University, Yichang, Hubei, China; 3 National Field Scientific Observation and Research Station for Landslides in the Three Gorges Area of Hubei Yangtze River, Yichang, Hubei, China; 4 Xingshan County Land Consolidation Center, Yichang, Hubei, China; Guizhou University, CHINA

## Abstract

After rainfall-induced landslides enter the creep deformation stage, timely mitigation is often challenging, making reasonable and effective early warning critical for reducing disaster losses. This study focuses on the Tanjiawan landslide, introducing the concept of “a single rainfall process” to characterize the rainfall process affecting landslide deformation. Based on a detailed analysis of deformation characteristics such as displacement and displacement rate under rainfall, the least squares method is used to identify the “failure inflection point” and “stable inflection point” on the “step-like” deformation curve to determine the accelerated deformation interval. This approach further establishes the antecedent rainfall threshold (*Pe*), current rainfall (*P*), and displacement rate threshold (*V*). Subsequently, a refined dynamic early warning model is developed by integrating the function *F*(*V*, *P*, *Pe*) with a Logistic regression model. The findings indicate: (1) The deformation of the Tanjiawan landslide is closely correlated with rainfall processes, with cumulative displacement curves exhibiting distinct “step-like” characteristics and displacement rates showing a “lagged attenuation” phenomenon. (2) Finer monitoring cycles enable more precise capture of dynamic landslide deformation, resulting in more reliable displacement rate thresholds. (3) The landslide early warning model can dynamically adjust monitoring cycles based on the evolutionary characteristics of deformation stages, achieving adaptive monitoring optimization.

## Introduction

Landslides, as geological hazards triggered by factors such as rainfall and earthquakes, present significant challenges in prediction and mitigation during the creep deformation stage due to their dynamic complexity and nonlinear evolution characteristics [1,2]. Globally, frequent landslide events pose severe threats to human life, property, and the ecological environment, necessitating scientific approaches to reduce disaster losses. Early warning systems are critical for disaster prevention and mitigation, providing risk alerts before a landslide occurs. However, traditional static threshold methods struggle to capture the dynamic deformation characteristics induced by rainfall, resulting in limited prediction accuracy [3–8]. Consequently, refined early warning models based on real-time monitoring data and dynamic analysis have become a key direction for enhancing landslide disaster prevention capabilities [9–13].

Rainfall is widely recognized as a primary trigger for landslide deformation and instability [14–16]. Landslide activity often peaks during the flood season when rainfall intensity surges [17–20]. Rainfall characteristic parameters—such as intensity, duration, cumulative rainfall, and antecedent rainfall—significantly influence landslide triggering and evolution [21–31]. Rainfall intensity and duration (*I-D*) thresholds are commonly used to predict landslide occurrence and warn of potential risks, but their effectiveness is constrained by unknown factors such as groundwater content [32–35].

In recent years, the integration of high-temporal-resolution monitoring technologies and advanced data analysis methods has been driving the field of landslide early warning towards dynamic and refined development [36–39]. At the data acquisition level, technologies such as high-frequency GNSS and new-generation InSAR have enabled continuous, millimeter-level, hourly, or even minute-level monitoring of surface deformation, providing critical data support for analyzing transient responses and cumulative evolutionary patterns of displacement during rainfall processes [40,41]. In terms of model development, machine learning methods like support vector machines can automatically identify complex nonlinear precursors to landslide instability from multi-source monitoring data. Meanwhile, emerging techniques such as physics-informed neural networks enhance the interpretability and predictive robustness of models by embedding soil-rock mechanical constitutive relationships into data-driven frameworks [42–43]. Nevertheless, key challenges and cutting-edge directions in the field remain, including how to achieve real-time assimilation and fusion of multi-source heterogeneous monitoring data and how to establish early warning mechanisms capable of adaptively adjusting according to the deformation stage of a landslide. In-depth exploration of these directions will lay a crucial technical foundation for developing next-generation intelligent landslide early warning systems.

This study focuses on the Tanjiawan landslide, analyzing the impact of rainfall on landslide deformation. From 2020 to 2021, the landslide exhibited significant “step-like” deformation patterns at multiple monitoring points, with peak deformation rates lagging behind peak rainfall. By analyzing thresholds for current rainfall (*P*), antecedent rainfall (*Pe*), and displacement rate (*V*) under different monitoring cycles, it was found that shortening the monitoring cycle (from 24 hours to 1 hour) enables the capture of subtle deformation fluctuations influenced by external factors such as rainfall. Based on these findings, a dynamic monitoring and early warning model was developed by integrating the function *F*(*V*, *P*, *Pe*) with a Logistic regression model [44–47]. This model dynamically adjusts monitoring cycles by analyzing factors such as displacement rate, current rainfall, and antecedent effective rainfall, enabling real-time adaptation of early warning strategies across different deformation stages.

### Regional setting

#### Geological setting.

**Fig 1:**
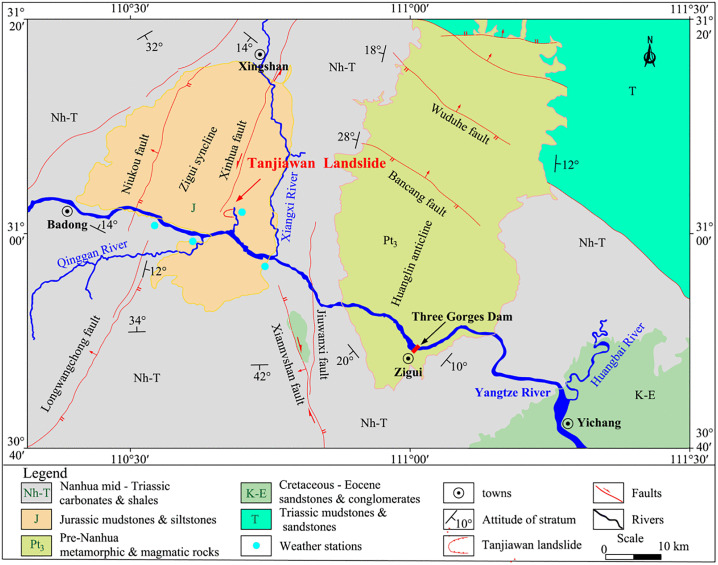
Landslide location map with geological formation distribution.

The Tanjiawan landslide is located in Shangba Village, Shuitianba Township, Zigui County, Yichang City, Hubei Province, China (Fig. 1), on the right bank of the Zhaixi River, a tributary of the Yangtze River. The Zigui syncline is characterized by a symmetrical S-shaped axis, with the axis trending approximately north-south, while the southern part is nearly east-west. The dip angle of the S(E) limb exceeds 30°, while the N(W) limb ranges between 16–30°. The core of the syncline consists of the Upper Jurassic Penglaizhen Formation, forming an annular basin in Zigui County with an axial length of 47 km. The landslide is situated in the core of the Zigui syncline, where folds and faults are well-developed, and the rock strata generally dip toward the northeast. Influenced by regional tectonics, north-northeast and near east-west trending fractures are prominently developed in the strata.

### Geomorphological features of Tanjiawan landslide

The Tanjiawan landslide is located on the northern slope of a high mountain, with a terrain resembling a concave armchair-shaped groove. The elevation at the mountain top ranges from approximately 600–650 m, while the foot of the slope, situated on the right bank of the Guizhou River, has an elevation of about 168–170 m. The landslide exhibits a “tongue-shaped” morphology with a zigzag profile, an average width of 295 m, a maximum longitudinal length of 440 m, an average thickness of 25 m, an area of approximately 1.13 × 10⁵ m^2^, and a volume of about 2.825 × 10⁵ m^3^. The main sliding direction is 68°, classifying it as a large-scale, deep-seated soil landslide. The rear boundary of the landslide is defined by a steep bedrock cliff on the southwestern slope top, with elevations ranging from 328–360 m, extending nearly north-south along the cliff, which is 10–15 m high. The right boundary is demarcated by a southeast-trending gully with an orientation of approximately 64°, where bedrock is exposed at certain points along the gully bottom. The left boundary is defined by a small northwest-trending ridge and its underlying depression, controlled by the interface between rock and soil and deformation points. The frontal shear outlet is located above the bedrock exposure in the Guizhou River channel, with the left side at an elevation of 195–200 m and the central and right sides at 168–170 m.

The landslide body primarily consists of silty clay with gravel, with a soil-to-rock ratio ranging from approximately 7:3–9:1. The gravel component comprises argillaceous siltstone and quartz sandstone, with diameters typically ranging from 1–5 cm, and larger fragments reaching 8–20 cm (Fig 3a). The slide zone soil is mainly composed of gravelly silty clay, reddish-brown to brownish-yellow in color, wet, and in a soft plastic to plastic state, with a soil-to-rock ratio of 9:1 and a thickness of approximately 0.2–0.4 m (Fig 3b and Fig 3c). The slide bed is composed of the Upper Jurassic Penglaizhen Formation, primarily consisting of mudstone, sandstone, quartz sandstone, and muddy conglomerate, which are prone to weathering (Fig 3d and Fig 3e).

**Fig 2:**
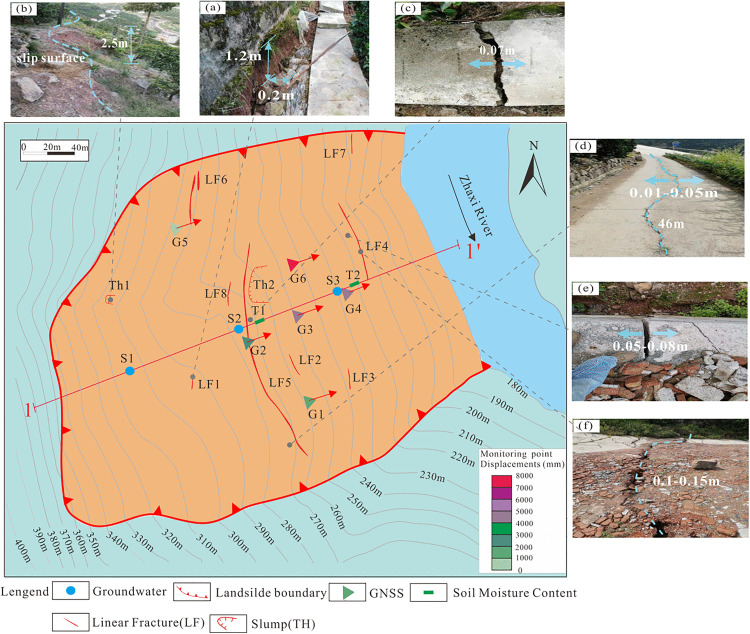
Monitoring point location plan and landslide planar morphology diagram.

### Macro-deformation features

From 2018 to 2021, the Tanjiawan landslide underwent progressive creep deformation under the influence of continuous heavy rainfall, with the deformation range gradually expanding from the middle-front to the middle-rear and both sides. In July 2018, multiple transverse tensile cracks appeared on the slope, such as Linear Fracture 1 (LF1) (Fig 2a) in the middle of the landslide, with an orientation of 165°, a length of approximately 135 m, a width of 5–20 cm, and a vertical displacement of about 120 cm.Influenced by rainfall during the flood season, subsequent deformations occurred in August 2018 and during the 2019 flood season, including multiple collapses and footpath deformations, such as the typical Slump 1 (TH1)in the middle of the slope (Fig 2b), with a volume of approximately 1,800 m^3^. Typical footpath deformations (Fig 2c) resulted in tensile cracks approximately 1–1.5 m long and 3–7 cm wide. From June to October 2020, landslide deformation intensified further, with five new Linear Fractures (LF3–LF7, ranging from 25–180 m in length with a maximum vertical displacement of 1.3 m) and the TH3 Slump forming in the middle-rear to middle-front sections. In the third quarter of 2021, heavy rainfall caused significant cracking in the frontal highway and newly constructed retaining walls (Fig 2e and Fig 2f), indicating the continued expansion of landslide deformation under rainfall influence. The specific locations of Linear Fractures LF1–LF7 and Slumps TH1–TH3 are shown in Fig 2.

### Monitoring results

The monitoring network for the Tanjiawan landslide was established in 2010 and fully completed in 2016, forming a comprehensive system focused on displacement, rainfall, groundwater, and moisture content. The system primarily includes six GNSS surface displacement monitoring devices, three groundwater level monitors (one each at the front, middle, and rear of the landslide), and two moisture content monitors (deployed in areas with intense deformation). The specific locations of all equipment are detailed in Fig 2.

### Groundwater

The pore-water pressure dynamics at monitoring points S1, S2, and S3 exhibited significant differences (Fig 4), primarily governed by the combined effects of rainfall and seasonal variations. Among them, the water level at point S1 displayed the most intense fluctuations, with a maximum single-event amplitude of 28.4 meters, demonstrating high synchrony with intense rainfall events. During the summer flood season, sustained and concentrated heavy rainfall served as the direct driver for its rapid rise and sustained high levels. For instance, in late July 2020, the cumulative effect of a series of rainfall events (28.8 mm on July 22 and 26.4 mm on July 24) directly prompted the water level to reach a high of 296.6 meters on July 26. More notably, before the water level peaked at 298.5 meters on August 27, 2021, the study area experienced extreme heavy rainfall, including 63.2 mm on August 24 and 24.0 mm on August 25, clearly illustrating the strong driving effect of major rainfall events on the water level at S1. Conversely, during the dry winter period (when the water level dropped to its annual minimum of 267.5 meters on January 10, 2021, preceded by virtually no rainfall), the water level receded to baseline fluctuations due to the interruption of recharge. This rapid response pattern of “rising with rain and falling with clearing,” coupled with an annual variation of 27.5 meters, indicates excellent connectivity between point S1 and atmospheric precipitation.

In contrast, the water level at point S2 exhibited much smaller fluctuations (an annual range of only about 2 meters), with its response to rainfall showing a distinct lag and attenuation effect. For example, the water level began to rise from 253.4 meters on June 24, 2020, which was a delayed response to consecutive rainfall events from June 21–23 (11.4 mm, 8.2 mm, and 7.2 mm, respectively), and remained elevated thereafter. The seasonal decline in its water level was also more gradual, not receding until after October 2, indicating a relatively slower drainage process. The fluctuations in 2021 were even more subdued; starting from the low in January, the water level gradually climbed under the sustained influence of concentrated rainfall, such as 39.0 mm on August 12 and 63.2 mm on August 24, eventually reaching its annual maximum of 255.4 meters on November 25. This delayed and smoothed dynamic presents a sharp contrast to the rapid, pulse-like response observed at point S1.

In contrast to S1 and S2, the groundwater level at monitoring point S3 remains relatively stable during the observation period, with fluctuations less than 5 m and no significant correlation with rainfall events. A notable water level spike occurred on June 29, with a single-day increase of 1.2 m, reaching a peak of 204.415 m, after which the level generally stabilized near this elevation. This is primarily attributed to recharge from surface water on the rear slope and groundwater from other aquifers.

### Soil water content

During the monitoring period from May 2020 to November 2021, the soil moisture content at 1.0 m depth in the study area exhibited distinct spatiotemporal variations in response to rainfall inputs. The consistently higher soil moisture recorded at monitoring point T2 compared to T1 reflects the significant influence of water supply from the rear section to the front (Fig. 5).

**Fig 3:**
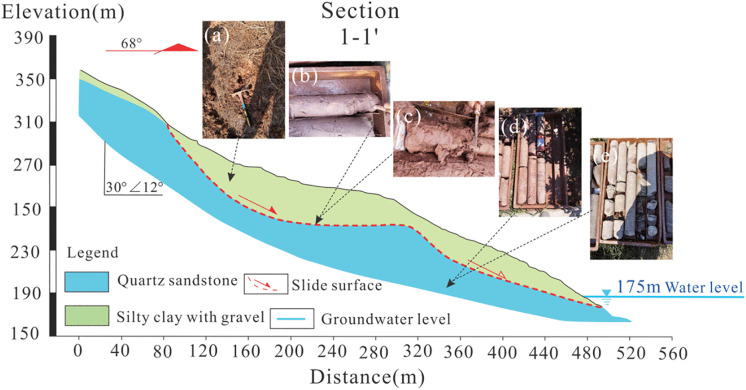
Profile 1−1’ and photographs illustrating the characteristics of the slide mass, slip zone, and slip bed.

**Fig 4:**
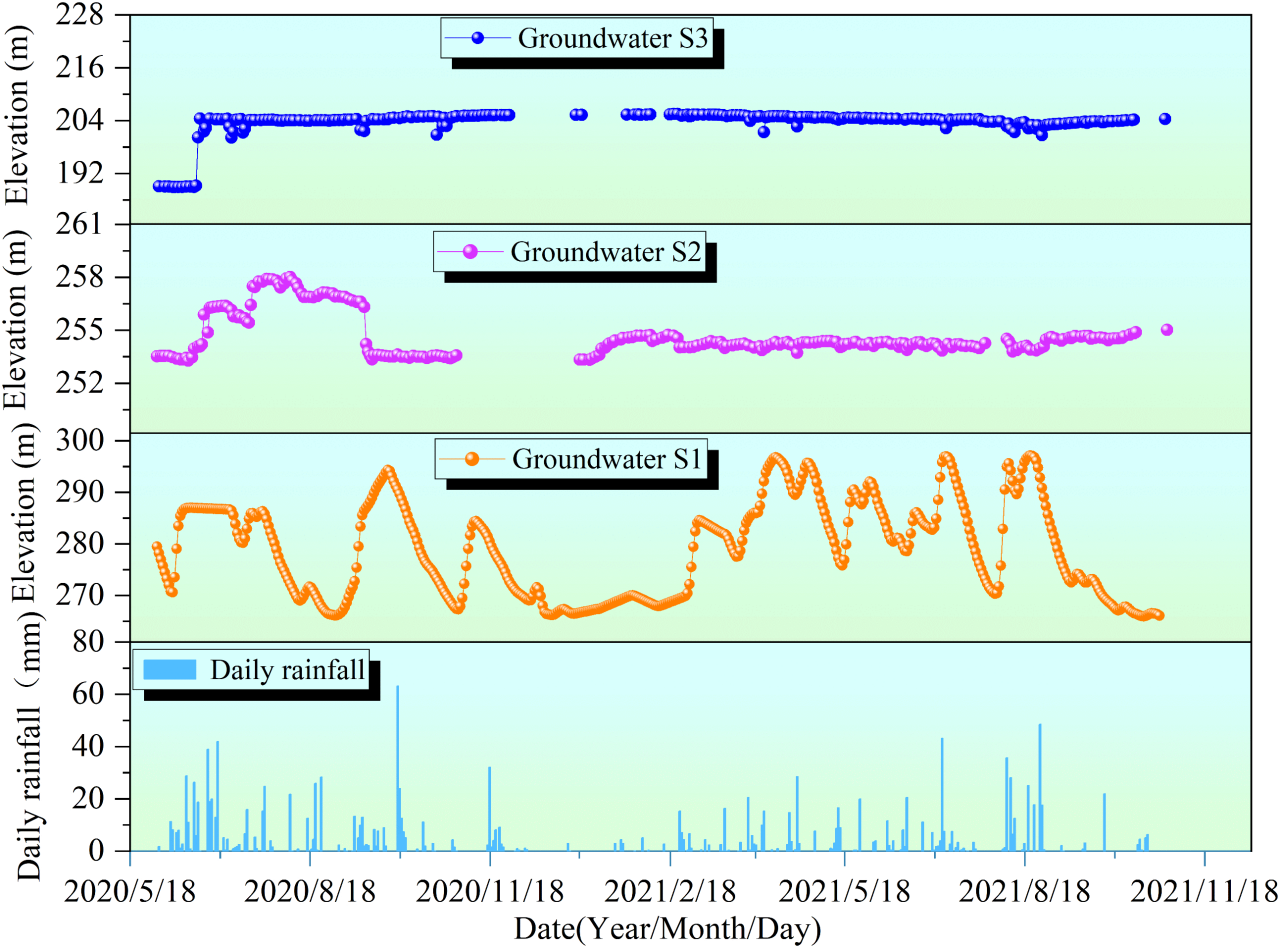
Relationship curves between groundwater levels (S1, S2, S3) and rainfall.

**Fig 5:**
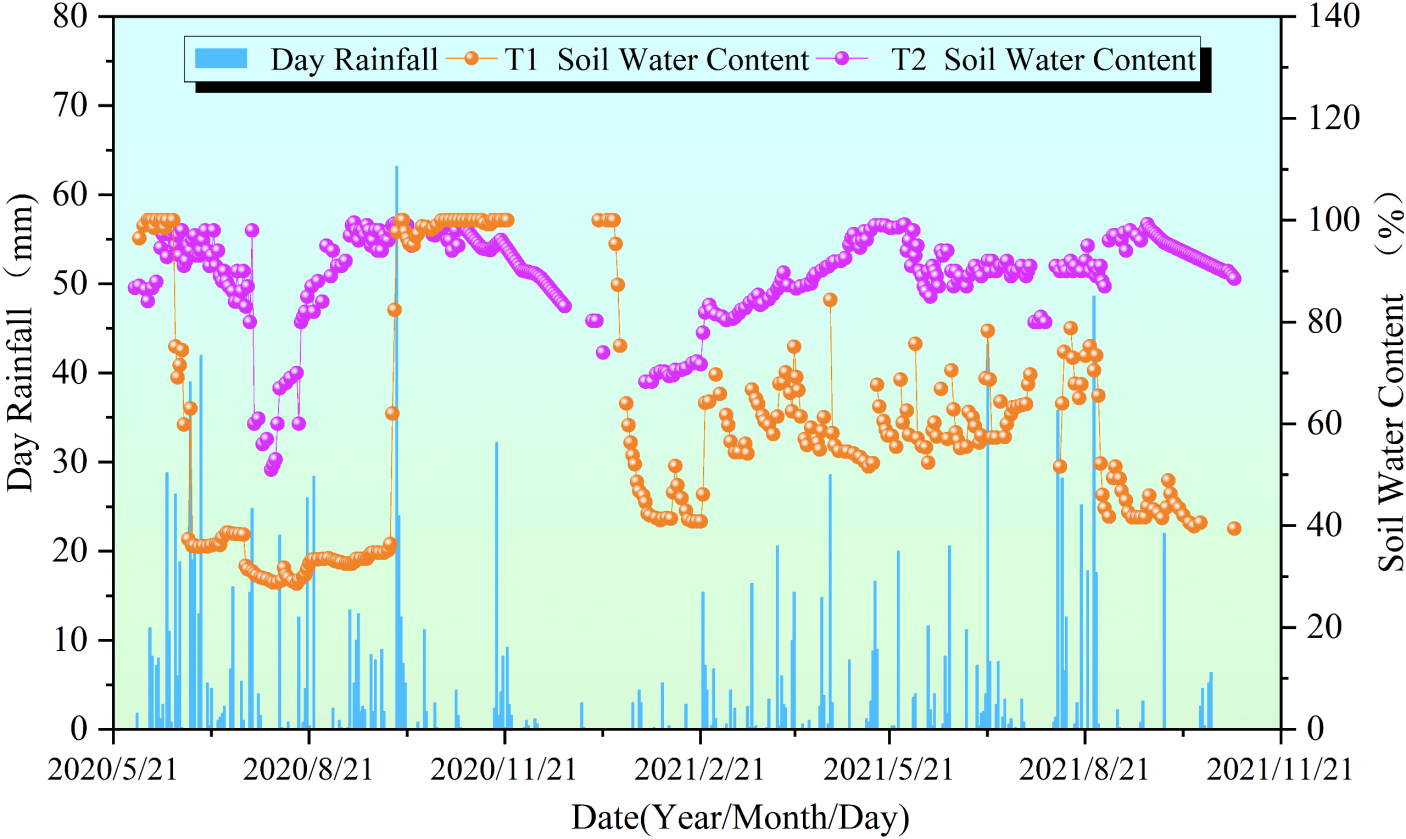
Relationship between soil moisture content (T1, T2) and rainfall.

In terms of temporal dynamics, the sharp decrease in moisture content at T1 from saturation to below 40% after June 24, 2020, was directly linked to the relatively low cumulative rainfall of approximately 40 mm in July. In contrast, point T2 remained saturated throughout the same period due to sustained lateral recharge. Subsequently, during the concentrated rainfall period from August to September (including a heavy rainfall event of 63.2 mm on August 24), the moisture content at T1 recovered to stable levels following adequate replenishment.

Data from 2021 further confirmed this pattern: the moisture content at T1 dropped to 39.6% during the dry period in January (monthly rainfall about 10 mm), fluctuated between 40% and 80% during the rainy season from April to July (including 26.0 mm of rainfall on May 2), and then showed a declining trend again after August when rainfall decreased. Correspondingly, starting from 68% at the end of January, point T2 reached saturation under the influence of spring rainfall (cumulative rainfall exceeding 80 mm in April) and did not transition to unsaturated conditions until the dry season began in November.

### Cumulative displacement

Since 2016, six GNSS monitoring stations have been deployed on the surface of the Tanjiawan landslide to collect data. Fig 7 clearly illustrates the cumulative displacement variations at each monitoring point from May 2020 to November 2021. All stations exhibited typical “stepped” deformation characteristics, where displacement increased rapidly during periods of intense rainfall but remained relatively stable during dry spells.

**Fig 6:**
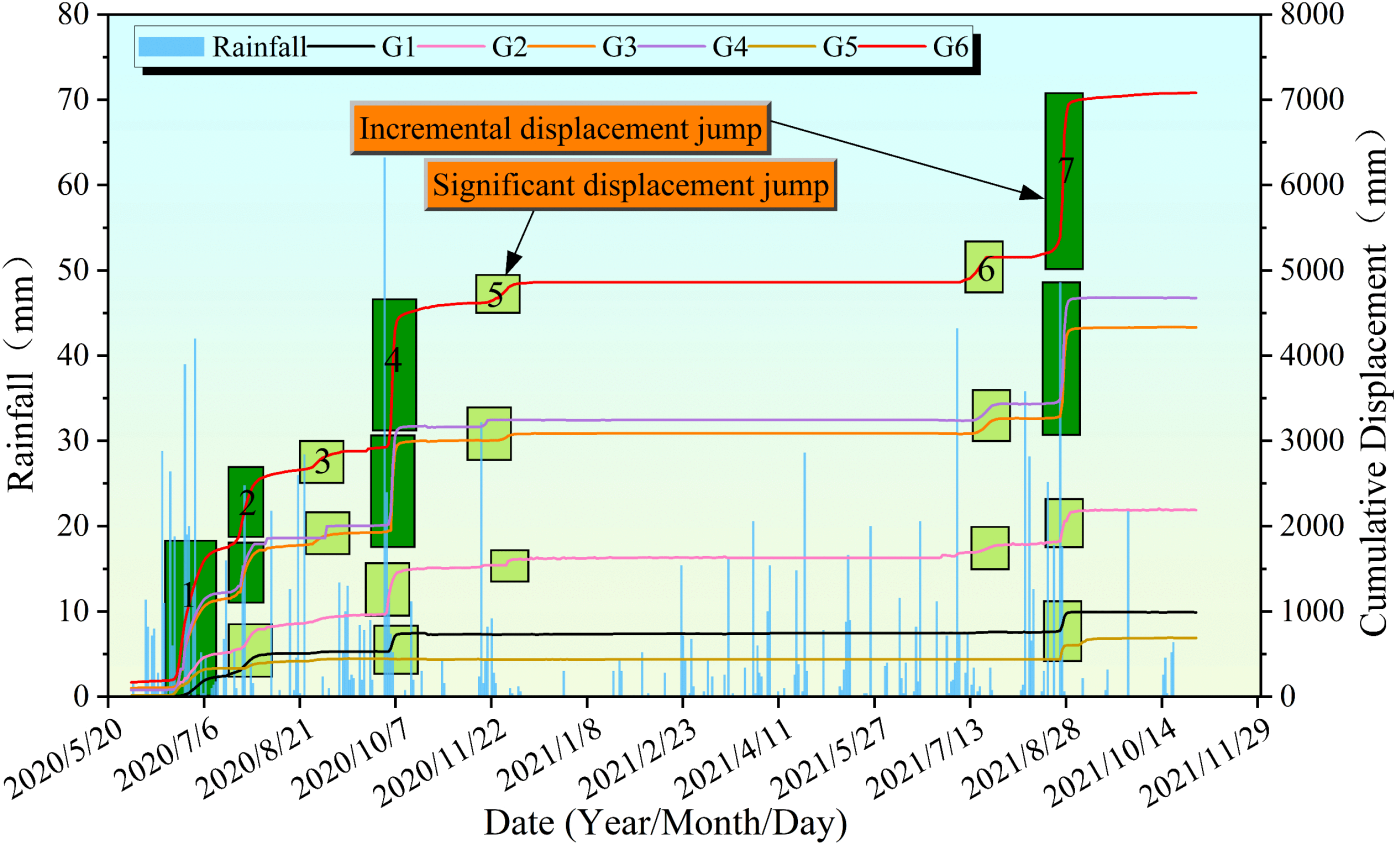
Cumulative displacement-rainfall curve.

In the second half of 2020, the study area experienced multiple episodes of concentrated rainfall, particularly on August 24 (63.2 mm in a single day) and throughout September. During this period, the cumulative displacements at stations G1 to G6 reached 691.34 mm, 1633.6 mm, 3089 mm, 3243.3 mm, 321.5 mm, and 4862.9 mm, respectively. Notably, the five distinct acceleration phases observed at station G6 closely corresponded with these heavy rainfall events.

In 2021, displacement growth at all stations slowed during the first half of the year when rainfall was relatively scarce, with cumulative displacements of 256 mm (G1), 564.82 mm (G2), 1244.99 mm (G3), 1432.85 mm (G4), 254.03 mm (G5), and 2221.77 mm (G6). However, in the second half of the year, triggered by heavy rainfall events such as those on August 12 (39.0 mm) and August 24 (63.2 mm), all monitoring points exhibited two episodes of stepped deformation of varying magnitudes. Among them, station G6 showed the most pronounced response, with its stepped increases aligning closely in timing with the major rainfall events. These observations collectively demonstrate a clear triggering relationship between concentrated heavy rainfall and the stepped displacement patterns of the Tanjiawan landslide.

The relatively flat segment observed in the cumulative displacement curve from December 2020 to June 2021 can be primarily attributed to distinct characteristics in rainfall intensity distribution. We selected the period from the second half of 2020 (2020/06/01–2020/12/31) and the first half of 2021 (2021/01/01–2021/07/01) as the contrasting time intervals, with the former corresponding to a period of significant step-like deformation and the latter to a period of moderate deformation. The comparison of key rainfall parameters is presented in the following Table 1:

**Table 1:** Rainfall parameters.

Time Period	Average Daily Rainfall (mm)	Number of Days with Daily Rainfall >10 mm	Total Rainfall (mm)
2020/06/01-2020/12/31	3.45	25	739.4
2021/01/01-2021/07/01	1.83	8	334.2

From the Table 1, it can be observed that the total rainfall in the second half of 2020 was approximately 2.2 times that in the first half of 2021. The average daily rainfall was about 88% higher, and the number of days with daily rainfall exceeding 10 mm was more than three times greater (25 days vs. 8 days) in the latter period. These differences indicate that the rainfall in the second half of 2020 was more intense and extreme events occurred more frequently, making the landslide body more susceptible to accelerated deformation. In terms of displacement monitoring data, five distinct step-like deformation events occurred in the second half of 2020, resulting in a staircase-like upward trend in the cumulative displacement curve. In contrast, almost no step-like deformation occurred in the first half of 2021, with the cumulative displacement showing a gentler increase (less than half of the increase observed in the previous period). This trend is highly correlated with the rainfall parameters: cumulative displacement is primarily influenced by the accumulation of antecedent effective rainfall, while step-like deformation is more easily triggered by single-day high-intensity rainfall (>10 mm). In the first half of 2021, there were only eight days with daily rainfall exceeding 10 mm, and these events were relatively dispersed (with no consecutive high-intensity events), leading to a relatively mild stress adjustment process in the landslide body and no significant cumulative damage.

Specifically, the relatively flat segment observed in the cumulative displacement curve between December 2020 and June 2021 can be attributed to significant differences in rainfall intensity distribution. Although the total cumulative rainfall during this period was comparable to that of intervals that resulted in distinct step-like deformation, the rainfall events were characterized by longer duration and lower intensity. This stands in stark contrast to the short-duration, high-intensity rainfall events (such as the 63.2 mm daily rainfall on August 24, 2020) that triggered notable step-like displacements. The low-intensity, prolonged rainfall reduced infiltration efficiency, causing a large portion of the precipitation to be lost through surface evaporation and retention in shallow soils, thereby failing to generate the deep, concentrated infiltration required to activate the sliding zone. Consequently, the displacement response of the slope remained in a mild state throughout this period.

**Fig 7:**
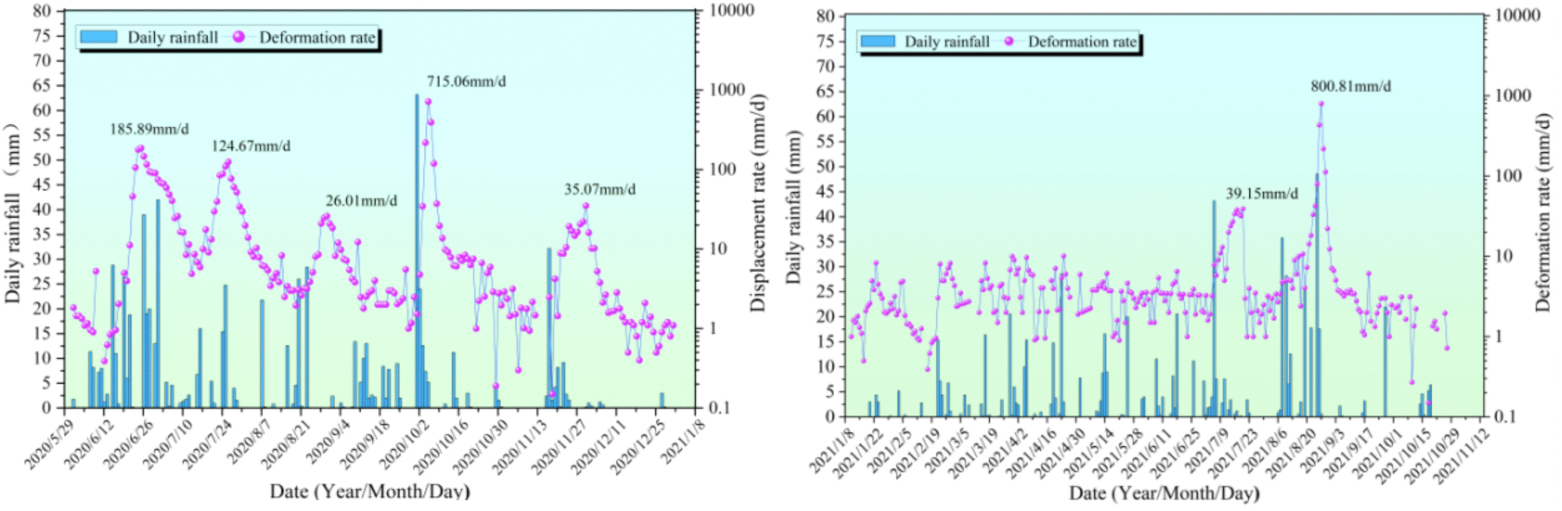
Curve of G6 displacement rate versus rainfall.

The displacement rate-rainfall curve for G6 from May 2020 to November 2021 is shown in Fig 7. During this period, seven peak displacement rates were observed, corresponding to seven distinct step-like deformation intervals in the G6 displacement curve. The maximum peak rate of 800.81 mm/d occurred at the end of August 2021, while the minimum peak rate of 26.01 mm/d was recorded at the end of August 2020. In the second half of 2020, the G6 displacement rate curve exhibited five peaks, which precisely coincided with the step-like deformation intervals in the G6 displacement curve. The specific peak rates were 185.89 mm/d, 124.67 mm/d, 26.01 mm/d, 715.06 mm/d, and 35.07 mm/d. Among these, the maximum peak rate of 715.06 mm/d occurred on October 7, 2020, within the fourth accelerated deformation interval of the G6 displacement curve.

In 2021, the amplitude of G6 displacement rate fluctuations was reduced compared to the second half of 2020, but two significant peak rates were still observed, corresponding to the sixth and seventh deformation intervals of the G6 displacement curve. These peak rates were 39.15 mm/d and 800.81 mm/d, respectively, and their occurrences were highly correlated with rainfall. Although the overall displacement rate fluctuations in 2021 were less intense than those in the second half of 2020, significant peaks in the G6 displacement rate were still observed during periods of heavy rainfall.

**Fig 8:**
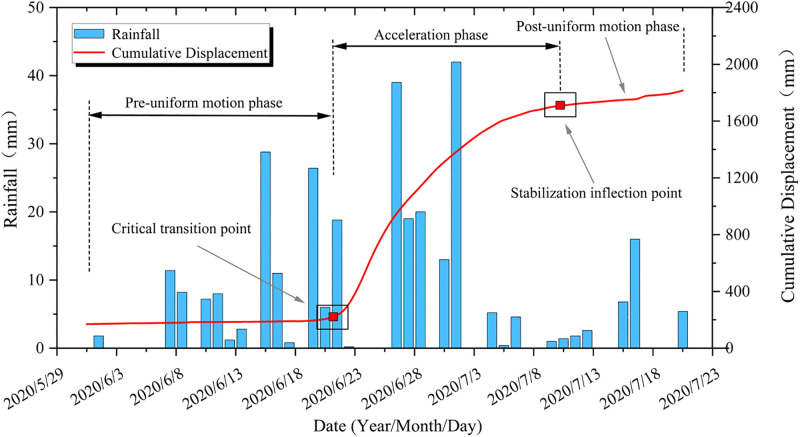
The first step deformation-displacement curve.

Fig 6 shows that all monitoring points exhibited varying degrees of “step-like” deformation. To define the deformation process of the step-like intervals, this study adopts the concepts of “mutation inflection point” and “stable inflection point” to delineate the step-like intervals. The step-like process can be divided into three stages: pre-constant velocity, acceleration, and post-constant velocity. The “mutation inflection point” marks the transition from pre-constant velocity to acceleration, while the “stable inflection point” indicates the transition from acceleration to post-constant velocity (Fig 8).

**Fig 9:**
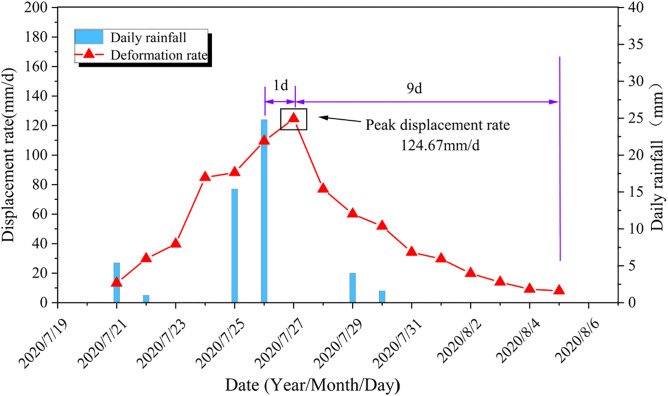
Hysteresis decay characteristic curve.

Fig 9 illustrates the relationship between rainfall and displacement rate at the G6 monitoring point during the second “step-like” deformation interval, revealing the characteristic of “lag-attenuation” in rainfall-induced deformation. For the Tanjiawan landslide, the “lag-attenuation” time T (=*∆T1* + *∆T2*) is approximately a constant value. This is manifested as follows: the peak displacement rate typically occurs T1 time (“lag time”) after the cessation of a rainfall event, generally ranging from 1 to 2 days. Additionally, after the peak displacement rate occurs, the displacement rate gradually attenuates until it returns to the normal deformation level of the landslide, with the “attenuation time” T2 being a fixed value. During the second step-like deformation interval, the landslide’s *∆T1* was 1 day, and *∆T2* was 9 days. Using the formula T (=*∆T1* + *∆T2*), the lag period of the landslide affected by rainfall is calculated to be 10 days.

## Methods

### Method for determining the acceleration interval of “Step-like” deformation

To determine the deformation acceleration intervals, landslide deformation data are extracted with a monitoring period of d. Exploration points are established centered on the midpoints of two consecutive monitoring periods, and multiple calculation points are set up. Linear regression coefficients are calculated for the data before and after the exploration points using the least squares method. At the calculation point corresponding to time *Ti*, linear regression is performed on n data points before and after Ti, and the regression coefficients are denoted as k_i_*(T*_*i*_) and k_r_*(T*_*i*_), respectively:


$$k_{i}\left(T_{i}\right)=\frac{n\sum_{i-n+\text{1}}^{i}S_{i}T_{i}-\sum_{i-n+\text{1}}^{i}S_{i}\sum_{i-n+\text{1}}^{i}T_{i}}{n\sum_{i-n+\text{1}}^{i}T^{\text{2}}-{\left(\sum_{i-n+\text{1}}^{i}T_{i}\right)}^{\text{2}}}$$ (1)

$$k_{r}\left(T_{i}\right)=\frac{n\sum_{i}^{i+n-\text{1}}S_{i}T_{i}-\sum_{i}^{i+n-\text{1}}S_{i}\sum_{i}^{i+n-\text{1}}T_{i}}{n\sum_{i}^{i+n-\text{1}}T^{\text{2}}-{\left(\sum_{i}^{i+n-\text{1}}T_{i}\right)}^{\text{2}}}$$ (2)

where *Si* represents the displacement monitoring value at time *Ti*.

*k*_*i*_*(T*_*i*_) and *k*_*r*_*(T*_*i*_) are the linear regression coefficients for the data preceding and following the exploration point Ti, respectively.

To accurately obtain *k*_*i*_*(T*_*i*_) and kr*(T*_*i*_), the regression coefficients are calculated for n = 2, 3, 4,..., n, resulting in *k*_*i*_*(2)(T*_*i*_*), k*_*r*_*(2)(T*_*i*_*), k*_*i*_*(3)(T*_*i*_*), k*_*r*_*(3)(T*_*i*_*), k*_*i*_*(4)(T*_*i*_*), k*_*r*_*(4)(T*_*i*_*),..., k*_*i*_*(n)(T*_*i*_*), k*_*r*_*(n)(T*_*i*_). Their weighted averages are then computed:


$$\overset{―}{k_{i}}\left(T_{i}\right)=\frac{\sum_{\text{2}}^{n}j\cdot k_{i}^{\left(j\right)}\left(T_{i}\right)}{\sum_{\text{2}}^{n}j}$$ (3)

$$\overset{―}{k_{r}}\left(T_{i}\right)=\frac{\sum_{j=\text{2}}^{n}j\cdot k_{r}^{\left(j\right)}\left(T_{i}\right)}{\sum_{j=\text{2}}^{n}j}$$ (4)

The first-order difference corresponding to Ti is calculated and denoted as *(T*_*i*_):


$$\Delta \left(T_{i}\right)=\overset{―}{k_{r}}\left(T_{i}\right)-\overset{―}{k_{i}}\left(T_{i}\right)$$ (5)

The second-order difference of the (T_i_) sequence is calculated and denoted as *2(T*_*i*_):


$$\Delta^{\text{2}}\left({\text{T}}_{\text{i}}\right)=\Delta \left({\text{T}}_{\text{i}}\right)-\Delta \left({\text{T}}_{\text{i}-\text{1}}\right)$$ (6)

The interval where *(T*_*i*_) and *2(T*_*i*_) are positive represents the starting point, termed the “failure inflection point.” The interval where *(T*_*i*_) and *2(T*_*i*_) are significantly negative represents the endpoint, termed the “stable inflection point.” The period between these two points constitutes the deformation acceleration interval. Through analysis of the cumulative displacement curve and Equations (1) to (6), information on the seven deformation acceleration intervals for the G6 monitoring point was obtained, as shown in Table 2.

**Table 2:** Information sheet of seven step deformations.

Step-like Deformation (n)	Step Interval	Displacement Increment (mm)	Average Displacement Rate (mm/d)
1	June 22, 2020 – July 11, 2020	1505.05	75.25
2	July 18, 2020 – August 3, 2020	819.93	48.16
3	August 28, 2020 – September 4, 2020	140.14	18.84
4	October 4, 2020 – October 13, 2020	1569.62	173.92
5	November 24, 2020 – December 4, 2020	181.46	16.50
6	July 9, 2021 – July 22, 2021	287.0	20.5
7	August 22, 2021 – September 1, 2021	1788.70	162.54

### Definition of “Rainfall Process”

**Fig 10:**
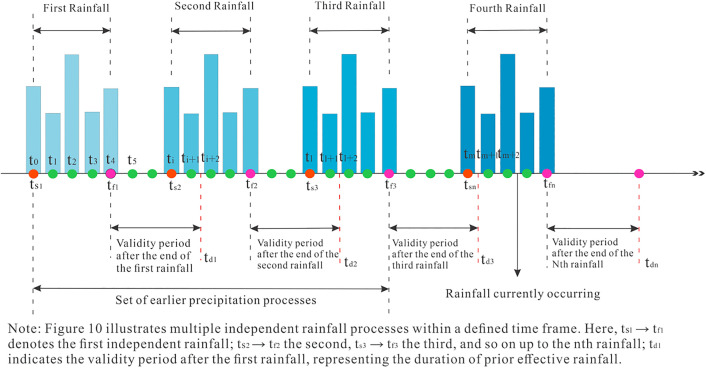
Schematic diagram of “A Rainfall Process”.

**Fig 11:**
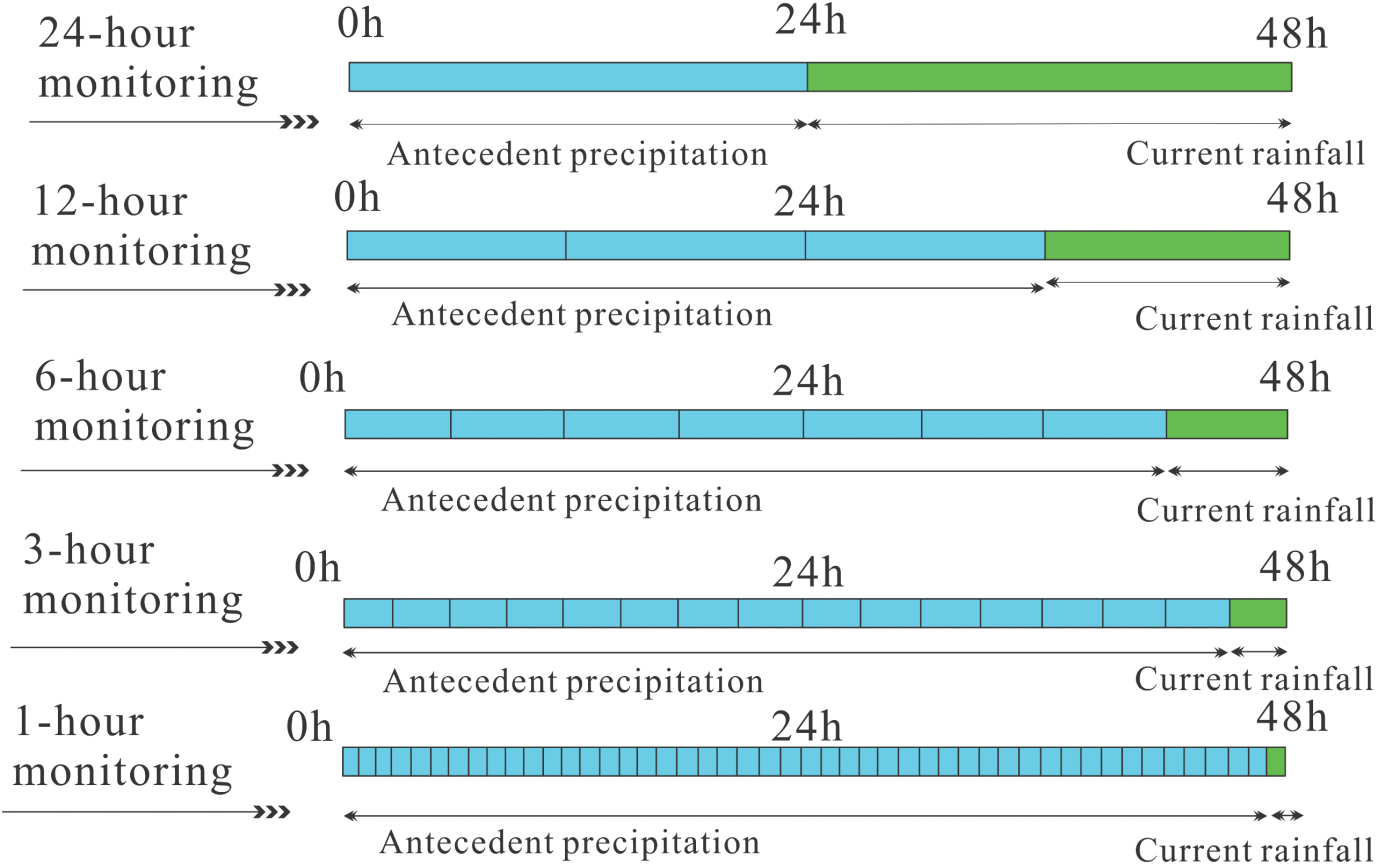
Schematic diagram of rainfall at different monitoring period.

The “rainfall process” (Fig 10) refers to treating multiple consecutive rainfall events as a single entity. The time interval Ti between the previous rainfall and the current rainfall is defined as the effective duration of the previous rainfall. When the time interval is less than or equal to Ti, the previous rainfall and the current rainfall together constitute a continuous rainfall process. When the interval exceeds Ti, the influence of the previous and current rainfall can be considered negligible. As the monitoring period shortens, the cumulative effect of the previous rainfall increases (Fig 11), with its influence becoming more significant. Meanwhile, the time window for the effect of the current rainfall narrows, primarily manifesting as the instantaneous triggering effect of short-term intense rainfall.

### Method for calculating effective rainfall

The Tanjiawan landslide transitions from stable creep to an unstable seasonal acceleration phase primarily triggered by sustained rainfall events rather than short-term intense rainfall. It exhibits a weak response to daily rainfall and is more significantly influenced by accumulated rainfall from previous events. According to the “single rainfall process” theory, the impact of prior rainfall on the landslide has a cumulative effect, which gradually attenuates over time. To quantify this effect, the concept of effective prior rainfall is introduced. This involves calculating the actual infiltrated rainfall that contributes to landslide deformation through a model, reflecting the dynamic characteristic of rainfall influence diminishing with time. This can be expressed using the following Equation (7):


$$Pe=\sum (R_{i}\times K_{i})$$

$$\text{0}\leq K_{i}\leq \text{1}$$ (7)

Where:

P_e_: Effective antecedent rainfall (mm), representing the rainfall that actually contributes to landslide deformation.

R_i_: Daily rainfall amount (mm) i days prior.

K_i_: Decay coefficient for the rainfall i days prior, which decreases over time (0 ≤ K_i_ ≤ 1), reflecting the diminishing influence of rainfall.

Clearly, the decay coefficient (K_i_) is central to this model. To objectively determine K_i_, it is derived inversely based on the “lag-decay” characteristic of the landslide displacement rate after rainfall ceases. The displacement rate influence degree (β) is defined as:


$$\beta =V_{n}/V_{max}$$ (8)

Where V_n_ is the displacement rate on the n-th day after rainfall cessation, and V_max is the peak rate during this process. β_n_ intuitively reflects the degree to which rainfall influence decays over time (β_max_ = 1).

To align with the physical reality that rainfall influence remains high until the peak is reached, the β sequence is adjusted by setting all pre-peak β values to 1. Using this adjusted β sequence, the Mitscherlich growth model is employed to fit the temporal variation of the decay coefficient K_i, as this model naturally describes processes of “diminishing returns.” Ultimately, the decay coefficient (K) for the Tanjiawan landslide can be expressed as a piecewise function of time (t):


K(t)=1t≤1 (9)

$$K(t)=\text{1}.\text{6}\text{7}\text{8}-\text{0}.\text{3}\text{2}\text{7}\times e^{-\text{0}.\text{1}\text{2}\text{6}t}t>\text{1}$$ (10)

**Fig 12:**
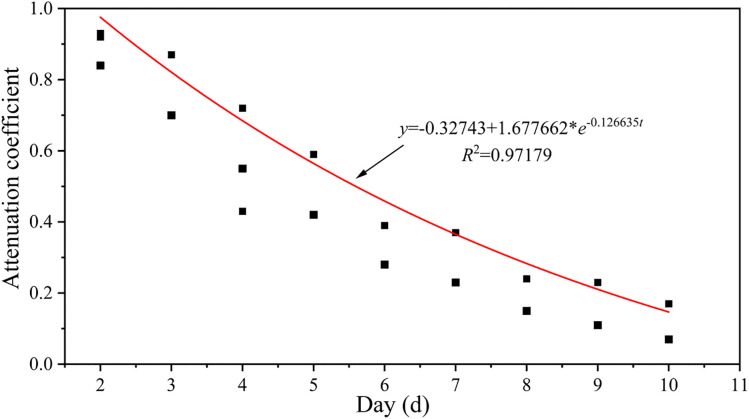
The “lag-decay” curve of the Tanjiawan landslide.

Through the analysis from Equations (7) to (10), the “lag-decay” curve for the Tanjiawan landslide is obtained (Fig 12), which clearly quantifies the dynamic process of how rainfall influence diminishes over time. This provides a key parameter for constructing a process-based early warning model.

### Refined analysis of rainfall and displacement rates across different monitoring periods

From May 2020 to November 2021, monitoring data for the Tanjiawan landslide revealed multiple significant step-like displacement events. These data highlight the sudden and intermittent rapid displacement characteristics during the landslide deformation process, with hourly displacement amounts reaching tens of times the daily displacement. To gain a deeper understanding of the evolutionary mechanisms of this nonlinear deformation process, there is an urgent need for higher-frequency and more refined threshold analysis.

For the refined analysis of step-like deformations, the step-like events with the smallest and largest displacement increments among the seven occurrences during the period from May 2020 to November 2021 were selected for analysis. Based on displacement monitoring data and rainfall records, combined with the calculation methods for deformation acceleration intervals and effective rainfall, the rainfall thresholds and displacement rate thresholds triggering deformation acceleration were analyzed under different monitoring periods (24h, 12h, 6h, 3h, and 1h). (The data in the table below represent the rainfall and displacement rates one day before and after the peak rate.)

Within the step-like deformation interval characterized by the minimal displacement increment, data from Table 3 reveals that the antecedent effective rainfall is the core driving factor controlling deformation evolution. Although the recorded rainfall was 0 mm during the monitoring window from August 28–30 across all periods, the antecedent effective rainfall remained at a high level of 19.3–44.8 mm, sustained by the continuous replenishment from cumulative rainfall of 54.4 mm during August 20–24. This hydrological process significantly influences slope stability through three mechanisms: First, the high antecedent effective rainfall within the 19.3–44.8 mm range ensures persistent moisture supply, maintaining the soil water content at 30%−40% at monitoring point T1, while point T2, influenced by rear-edge surface water recharge, reaches approximately 85%. This moisture-sustained high water content leads to an uneven distribution of pore water pressure within the sliding mass [48]. Second, the infiltration from the antecedent cumulative rainfall of 54.4 mm sufficiently saturates the rock and soil mass, not only significantly increasing the unit weight of the sliding body but also softening the slip zone soil through lubrication and physico-chemical effects, thereby reducing its shear strength. Third, the sustained antecedent rainfall enhances water-rock interactions, altering the rheological properties of the rock and soil mass and significantly increasing its creep capacity. These mechanically processes dominated by antecedent effective rainfall maintain high deformation activity in the landslide even without additional rainfall, specifically manifested in the 24h monitoring cycle with a displacement rate peak of 39.57 mm/d on August 29, lagging approximately one day behind the peak antecedent effective rainfall.

**Table 3:** Rainfall and displacement rates under different monitoring periods (step deformation with minimal displacement increment).

Monitoring Period (T_24/12/6/3/1_)	Rainfall Date (YYYY/MM/DD)	Current Rainfall (mm)	Effective Prior Rainfall (mm)	Displacement Rate (mm/24h,12h,6h,3h,1h)
T_24_=[0, 8]	2020/8/28	0	31.6	10.58
	2020/8/29	0	25.0	39.57
	2020/8/30	0	19.3	24.92
T_12_=[0, 15]	2020/08/28 00:00-12:00	0	44.8	5.9
	2020/08/28 12:00-24:00	0	39.1	7.68
	2020/08/29 00:00-12:00	0	36.7	22.65
	2020/08/29 12:00-24:00	0	31.6	20.49
	2020/08/30 00:00-12:00	0	29.5	16.51
	2020/08/30 12:00-24:00	0	23.0	6.91
T_6_=[0, 31]	2020/08/28 12:00-18:00	0	31.6	7.71
	2020/08/28 18:00-24:00	0	29.5	10.8
	2020/08/29 00:00-06:00	0	29.5	12.48
	2020/08/29 06:00-12:00	0	28.5	14.81
	2020/08/29 18:00-24:00	0	27.2	12.82
	2020/08/30 00:00-06:00	0	26.1	11.83
T_3_=[0, 63]	2020/08/28 18:00-21:00	0	32.4	4.96
	2020/08/28 21:00-24:00	0	31.6	5.01
	2020/08/29 00:00-03:00	0	30.3	5.27
	2020/08/29 03:00-06:00	0	29.8	5.21
	2020/08/29 06:00-09:00	0	29.5	5.36
	2020/08/29 09:00-12:00	0	29.1	5.64
	2020/08/29 12:00-15:00	0	28.2	5.14
	2020/08/29 15:00-18:00	0	27.5	4.68
	2020/08/29 18:00-21:00	0	26.7	4.42
	2020/08/29 21:00-24:00	0	26.0	3.41
	2020/08/30 00:00-03:00	0	26.5	4.55
	2020/08/30 03:00-06:00	0	25.2	3.51
T_1_=[0, 127]	2020/08/28 22:00-23:00	0	31.6	2.3
	2020/08/28 23:00-24:00	0	31.6	2.5
	2020/08/29 00:00-01:00	0	31.6	2.3
	2020/08/29 01:00-02:00	0	31.3	2.9
	2020/08/29 02:00-03:00	0	30.3	3
	2020/08/29 03:00-04:00	0	29.6	3.1
	2020/08/29 12:00-13:00	0	29.2	3.7
	2020/08/29 13:00-14:00	0	29.2	4.1
	2020/08/29 14:00-15:00	0	29.2	4.1
	2020/08/29 15:00-16:00	0	29.2	3.9
	2020/08/29 21:00-22:00	0	28.4	1
	2020/08/29 22:00-23:00	0	28.0	1.3
	2020/08/29 23:00-24:00	0	28.0	1.1
	2020/08/30 00:00-01:00	0	27.9	1.3
	2020/08/30 01:00-02:00	0	27.7	1.1

During the monitoring of the step-like interval, different monitoring periods reveal the dynamic characteristics of the displacement rate. The 24h monitoring period shows the displacement rate peaking at 39.57 mm/d on August 29, presenting an “increase-decrease” trend (10.58 mm/d → 39.57 mm/d → 24.92 mm/d). The 12h monitoring period identifies two increments and two decrements (peak of 22.65 mm/12h during August 29, 00:00–12:00), demonstrating a more detailed dynamic response. The 6h monitoring period further reveals four increments and multiple decrements (14.81 mm/6h during August 29, 06:00–12:00), capturing minor fluctuations during the decreasing process. The 3h monitoring period refines the increasing process into dynamic stages (5.36 mm/3h to 5.64 mm/3h during August 29, 06:00–12:00), reflecting nonlinear variation characteristics. The 1h monitoring period provides the highest resolution, demonstrating a stronger capability to capture minor deformations and refining the displacement rate increments and decrements into dynamic processes rather than simple monotonic changes.

These multi-temporal scale monitoring results indicate that the landslide deformation process exhibits similar fluctuation patterns across different time dimensions. Data from 24h to 1h monitoring show distinct hierarchical characteristics: longer monitoring periods outline the overall deformation trend, while shorter monitoring windows reveal increasingly finer fluctuations hidden beneath the macro trend. The deformation curves across these different time scales appear to follow a common rhythm, repeating the basic “acceleration-deceleration” cycle within their respective time dimensions. This cross-scale regularity suggests that the fundamental physical processes controlling landslide deformation operate continuously across different time scales, with their manifestations varying according to the observation scale. Therefore, short-period monitoring not only provides more accurate deformation early warning information but also helps us understand the unified patterns of landslide deformation across different time scales, offering crucial insights for establishing a multi-scale monitoring-based landslide early warning model.

Within the step-like deformation interval characterized by the maximum displacement increment, data from Table 4 indicate that antecedent intense rainfall is the critical factor triggering significant landslide deformation. Frequent rainfall occurred from June to August 2021, with the peak annual rainfall recorded at the end of August. Particularly, the continuous rainfall from August 20–27 (cumulative 109.2 mm) resulted in a notable cumulative effect of antecedent effective rainfall. This intense rainfall process significantly accelerated landslide deformation through the following mechanisms: First, persistent heavy rainfall infiltration maintained soil water content at 85%–90% at monitoring point T2 and 60%–70% at point T1. This ultra-high water content induced intense pore water pressure within the sliding mass. Second, the 48.6 mm rainfall on August 26 brought the rock and soil mass to near-saturation, substantially increasing the self-weight of the sliding body while significantly weakening the shear strength of the slip zone soil. Third, frequent rainfall events intensified water-rock interactions, leading to irreversible structural damage in the slip zone soil. These mechanically processes dominated by heavy rainfall resulted in severe landslide deformation, specifically manifested in the 24h monitoring cycle with a displacement rate peak of 800.81 mm/d on August 28, lagging approximately one day behind the peak antecedent effective rainfall.

**Table 4:** Rainfall and displacement rates under different monitoring periods (step deformation with maximum displacement increment).

Monitoring Period (T_24/12/6/3/1_)	Rainfall Date (YYYY/MM/DD)	Current Rainfall (mm)	Effective Prior Rainfall (mm)	Displacement Rate (mm/24h,12h,6h,3h,1h)
T_24_=[0, 11]	2021/8/27	17.6	82.6	403.62
	2021/8/28	0.6	70.7	800.81
	2021/8/29	0	71.3	234.00
T_12_=[0, 21]	2021/08/27 00:00-12:00	17.4	74.6	134.05
	2021/08/27 12:00-24:00	0.2	88.1	269.00
	2021/08/28 00:00-12:00	0.6	86.6	479.27
	2021/08/28 12:00-24:00	0.0	83.2	321.53
	2021/08/29 00:00-12:00	0.0	77.3	169.44
	2021/08/29 12:00-24:00	0.0	71.3	65.12
T_6_=[0, 43]	2021/08/27 12:00-18:00	0.20	88.1	106.93
	2021/08/27 18:00-24:00	0.00	87.5	178.42
	2021/08/28 00:00-06:00	0.6	86.6	232.09
	2021/08/28 06:00-12:00	0	85.4	247.19
	2021/08/28 18:00-24:00	0	79.5	101.03
	2021/08/29 00:00-06:00	0	77.3	96.29
T_3_=[0, 87]	2021/08/27 18:00-21:00	0	88.0	80.96
	2021/08/27 21:00-24:00	0	88.3	97.46
	2021/08/28 00:00-03:00	0	83.7	122.18
	2021/08/28 03:00-06:00	0	81.4	124.34
	2021/08/28 06:00-09:00	0	84.3	122.85
	2021/08/28 09:00-12:00	0	85.0	114.45
	2021/08/28 12:00-15:00	0	87.0	106.06
	2021/08/28 15:00-18:00	0	85.6	60.25
	2021/08/28 18:00-21:00	0	85.4	40.78
	2021/08/28 21:00-24:00	0	84.0	122.18
	2021/08/29 00:00-03:00	0	83.2	49.33
	2021/08/29 03:00-06:00	0	81.4	46.95
T_1_=[0, 263]	2021/08/27 22:00-23:00	0	88.3	32.4
	2021/08/27 23:00-24:00	0	88.3	34.7
	2021/08/28 00:00-01:00	0	88.3	35.7
	2021/08/28 01:00-02:00	0	88.2	37.2
	2021/08/28 02:00-03:00	0	88.0	37.0
	2021/08/28 03:00-04:00	0	87.9	39.7
	2021/08/28 12:00-13:00	0	85.4	33.6
	2021/08/28 13:00-14:00	0	85.3	31.7
	2021/08/28 14:00-15:00	0	85.3	30.8
	2021/08/28 15:00-16:00	0	84.0	28.3
	2021/08/28 21:00-22:00	0	83.2	22.4
	2021/08/28 22:00-23:00	0	83.2	19.8
	2021/08/28 23:00-24:00	0	83.2	20.9
	2021/08/29 00:00-01:00	0	83.2	15.4
	2021/08/29 01:00-02:00	0	83.1	15.6

During the monitoring of the maximum step-like interval, different monitoring periods clearly documented the complete process of accelerated landslide deformation. The 24h monitoring cycle revealed a sharp “surge-plummet” characteristic in the displacement rate (August 27: 403.62 mm/d → August 28: 800.81 mm/d → August 29: 234.00 mm/d), reflecting an abrupt response triggered by heavy rainfall. The 12h monitoring cycle identified two rapid increments and three decrements, with a peak of 479.27 mm/12h occurring during August 28, 00:00–12:00, revealing the phased variation characteristics of the deformation rate. The 6h monitoring cycle further captured three significant increasing fluctuations, with a peak of 247.19 mm/6h during August 28, 06:00–12:00, demonstrating a complex response pattern following rainfall attenuation. The 3h monitoring cycle refined the deformation process into more detailed dynamic stages. For instance, after reaching a peak of 124.34 mm/3h during August 28, 03:00–06:00, noticeable fluctuations occurred, dropping to 40.78 mm/3h (18:00–21:00) before rebounding to 122.18 mm/3h (21:00–24:00), indicating strong instability. The 1h monitoring cycle provided the most detailed deformation record, clearly illustrating instantaneous variation characteristics. For example, a peak of 39.7 mm/h occurred during August 28, 03:00–04:00, followed by a fluctuating decline until it decreased to 15.6 mm/h during August 29, 01:00–02:00.

These multi-temporal scale monitoring results indicate that large step-like deformations triggered by heavy rainfall exhibit more pronounced nonlinear characteristics and more complex dynamic response processes. Compared to the minimum step-like interval, the deformation rate in the maximum step-like interval is nearly 20 times higher and demonstrates more significant discontinuity and abruptness. Deformation curves under different monitoring periods show that under heavy rainfall conditions, landslide deformation not only exhibits cross-scale self-similarity but also distinct scale specificity: longer periods reflect the overall acceleration trend, while shorter periods reveal intense instantaneous fluctuations. This complex dynamic behavior is closely related to the cumulative effect of antecedent effective rainfall, the progressive failure process of the slip zone soil, and dynamic changes in pore water pressure. Therefore, under heavy rainfall conditions, multi-scale joint monitoring is particularly important for capturing abrupt deformation characteristics and identifying critical states, providing essential technical support for landslide early warning under extreme rainfall conditions.

### Key findings

Based on the comparative analysis of monitoring data from the minimum and maximum step-like deformation intervals, the core patterns of landslide deformation response to antecedent rainfall can be clearly identified: the deformation intensity shows a significant positive correlation with the antecedent effective rainfall, and the peak displacement rate generally lags approximately 24 hours behind the rainfall peak. Specifically, in the low-step interval shown in Table 2, when the antecedent effective rainfall was maintained between 19.3–44.8 mm, the triggered maximum displacement rate peak was 39.57 mm/d. In contrast, in the high-step interval shown in Table 3, when the cumulative antecedent effective rainfall reached 109.2 mm, the triggered maximum displacement rate peak sharply increased to 800.81 mm/d—a magnitude nearly 20 times greater. This stark contrast not only quantifies the rainfall and displacement conditions corresponding to different scales of step-like deformation but, more importantly, reveals the existence of a critical rainfall threshold range (approximately between 44.8 mm and 109.2 mm) that may trigger a sharp acceleration in landslide deformation. Furthermore, multi-period monitoring data indicate that the transition of the displacement rate from a relatively stable low-speed phase (1.0–4.1 mm/h in the T1 cycle) to a high-speed mutation phase (39.7 mm/h in the T1 cycle) is a key indicator of the landslide entering a dangerous state of accelerated deformation. These patterns systematically elucidate the deformation mechanism of rainfall-induced landslides, providing direct data support and a theoretical basis for the subsequent definition of rainfall thresholds and displacement rate thresholds for different warning levels.

### Rainfall and displacement rate thresholds

According to the data presented in Table 2 and Table 3, the Tanjiawan landslide exhibits high sensitivity to the accumulation of antecedent rainfall but a relatively weak response to current daily rainfall. To establish an early warning model, the average values of the antecedent rainfall and displacement rate corresponding to the minimum displacement increment interval were adopted as discriminant thresholds. This approach is based on statistical principles widely used in the field of landslide early warning, aiming to establish an initial identification benchmark that balances sensitivity, robustness, and a safety margin. The core of this methodology lies in using recorded instability events to calibrate critical conditions for future warnings, which is supported by established industry practices. For instance, Intrieri et al. (2012) [49] explicitly used statistical values of displacement rates from historical deformation phases as thresholds in the early warning system for the Rossi landslide in Italy, while Gariano et al. (2007) [50] systematically applied statistical methods to rainfall data from historical landslide events to define critical thresholds in global rainfall threshold research. Selecting the “average value” rather than extreme values helps smooth out monitoring errors, enhances the statistical robustness of the threshold, and aligns with the conservative principle of “rather false alarms than missed alarms” during the initial warning stage, providing a clear and reliable criterion for identifying the transition of the landslide from a stable state to an initial acceleration phase. Typically, thresholds defined by the minimum step interval are more suitable for identifying larger step-like deformations. Due to the weak influence of current rainfall on landslide deformation, it is difficult to define a threshold for current rainfall. Early warning and prediction can be achieved by using thresholds of antecedent rainfall and displacement rate to determine whether the landslide is about to deform.

1. Physical Basis: The average value of the minimum step-change interval (i.e., the shortest stable time between adjacent accelerated deformation events) has a physical significance in that it characterizes the typical time required for the landslide body to adjust its internal stresses and regain a quasi-static equilibrium after experiencing an external disturbance (such as rainfall). This value reflects the creep recovery characteristics of the landslide material and the memory effect of its critical state. When the actually observed stable interval is shorter than this typical recovery time, it means that the landslide body has not fully recovered from the previous disturbance before being subjected to a new one, resulting in the system’s accumulated damage not being fully dissipated, thereby significantly increasing the risk of overall instability.

2. Why Choose the Mean Over the Median or the Lower Envelope Limit: The selection of this average value over the median or the lower envelope limit is based on the following comprehensive considerations: Compared to the median, the mean is more sensitive to extreme short recovery periods, which are precisely the critical signals indicating the system’s most vulnerable state and closest proximity to instability. Using the mean allows for more proactive capture of these high-risk periods, aligning with the conservative principle of early warning systems that “it is better to have false alarms than to miss alerts.” Compared to the lower envelope limit, the lower limit represents historical extreme situations triggered by rare and special combinations of conditions (such as an unusually intense short-duration heavy rainfall). If used as a general threshold, it would be overly sensitive, leading to frequent false alarms under normal rainfall conditions and thereby reducing the credibility and usability of the warning system. In contrast, the mean is statistically more stable, representing the “normal” critical level of the system’s recovery capability across multiple disturbance events, achieving a better balance between warning sensitivity and system practicality. Choosing the “mean” over extreme values helps smooth out monitoring errors, enhances the statistical robustness of the threshold, and conforms to the conservative principle of the initial warning stage that “it is better to over-report than to miss reports,” providing a clear and reliable criterion for identifying the transition of the landslide from a stable state to an initial acceleration phase. This choice is widely adopted in geological landslide early warning systems because the average threshold provides a balanced representative description of slope behavior under typical rainfall conditions, while effectively identifying potential risks, significantly reducing false alarms caused by extreme values, and accounting for the inherent variability in geological, hydrological, and climatic contexts [51].The specific thresholds are presented in Table 5.

**Table 5:** Threshold values under different monitoring periods.

Monitoring Period	Effective Prior Rainfall Threshold	Displacement Rate Threshold
24h	25mm	18mm/24h
12h	26mm	13mm/12h
6h	27mm	8mm/6h
3h	28mm	5 mm/3h
1h	29mm	2mm/1h

**Fig 13:**
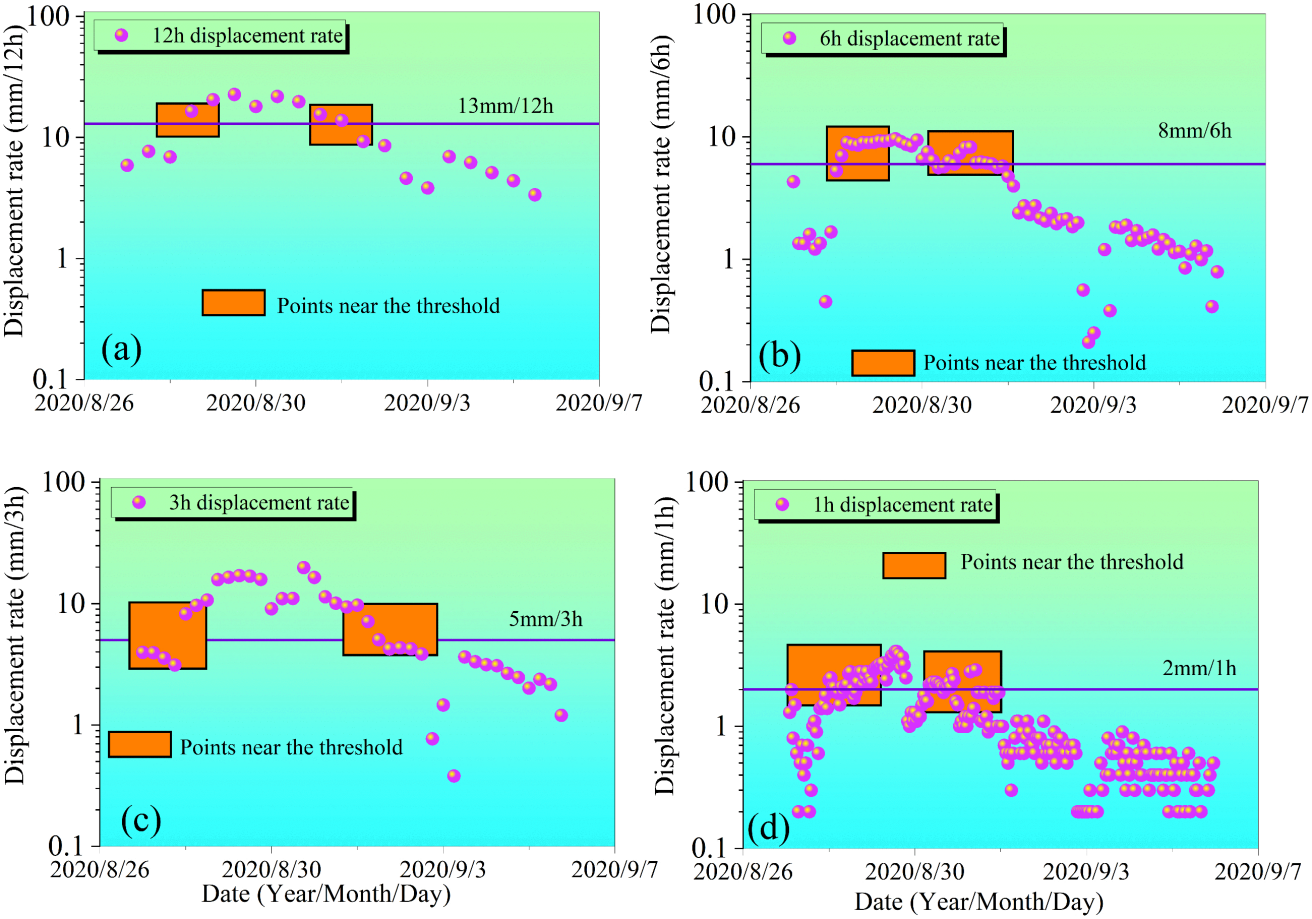
Displacement rate curve for the minimum step deformation.

**Fig 14:**
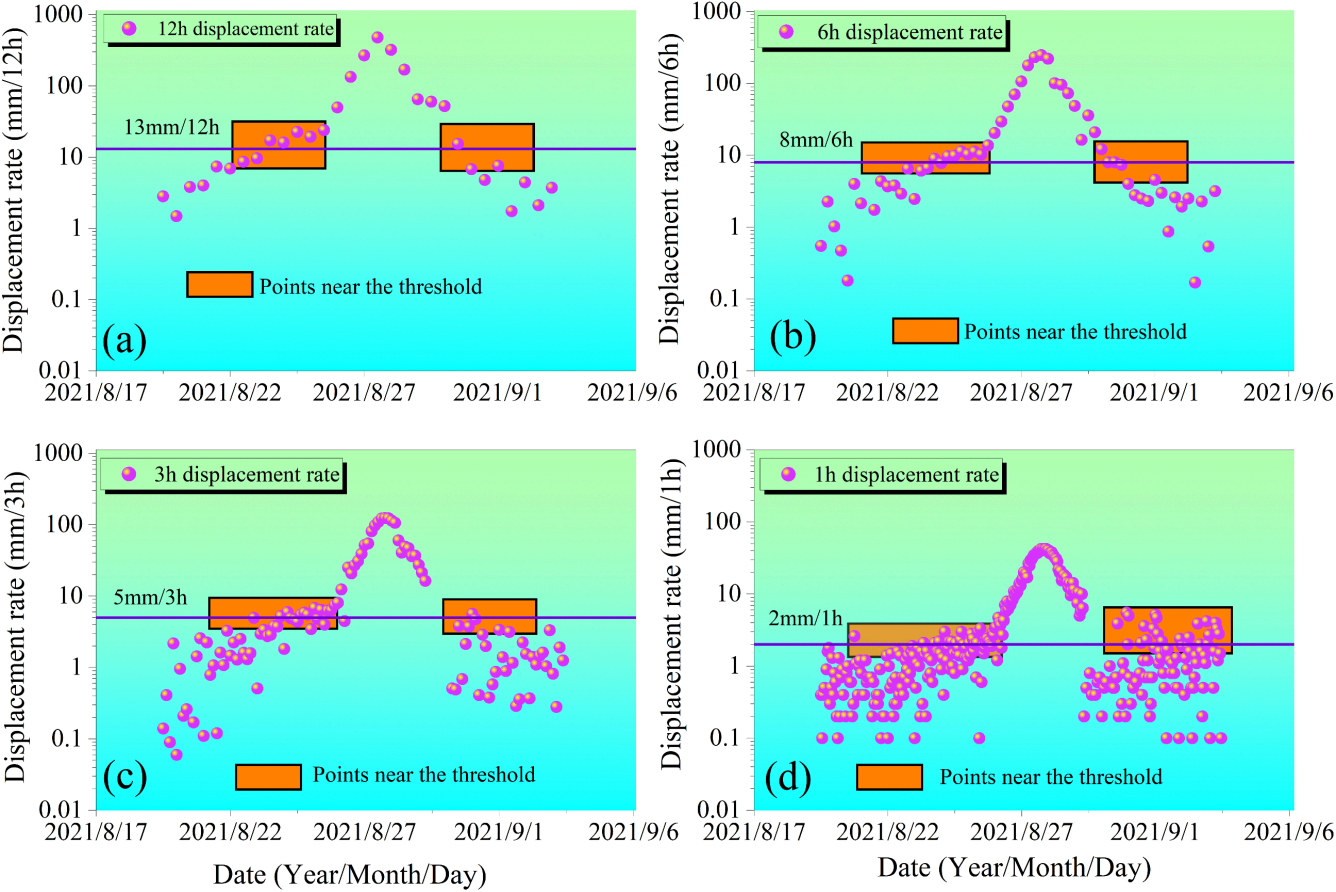
Displacement rate curve for the maximum step deformation.

Fig 13 and Fig 14 illustrate the displacement rate variation characteristics of the Tanjiawan landslide during the minimum and maximum displacement increment step-like deformation intervals under different monitoring periods (12h, 6h, 3h, and 1h). These figures also reveal the smoothing effect of long-period monitoring on data. As the monitoring period shortens from 12h to 1h, the number of data points near the displacement rate threshold curve (marked by orange boxes in Fig 13 and Fig 14) significantly increases, indicating a nonlinear improvement in the sensitivity of short-period monitoring to capture subtle deformations. This reflects that short-period monitoring can more precisely capture the dynamic changes in displacement rate, whereas long-period monitoring, due to the time-averaging effect, may smooth out minor fluctuations, leading to a reduction in data resolution.

### Establishment of the warning model

#### Classification of landslide warning levels.

**Fig 15:**
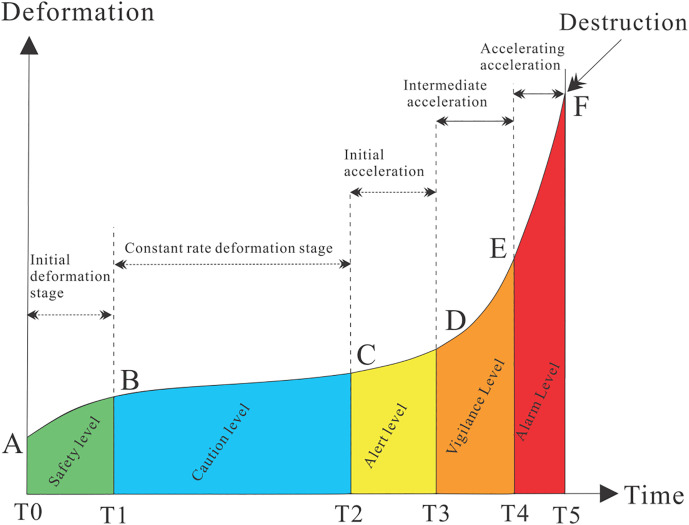
Landslide Early Warning Levels and Deformation Phase Classification.

Based on the warning classification system outlined in the Law of the People’s Republic of China on Emergency Response, geological disasters adopt a five-level warning system. However, clear quantitative standards for each warning level of geological disasters are still under development. The landslide deformation process is divided into multiple consecutive stages: the initial deformation stage, the constant-velocity deformation stage, and the accelerated deformation stage. The accelerated deformation stage is further subdivided into three sub-stages: initial acceleration, intermediate acceleration, and advanced acceleration (Fig 15).

### Logistic regression function model

Based on the combined assessment of displacement rate V, current rainfall P, and effective prior rainfall Pe, a function *F(V, P, Pe)* and a logistic regression model are introduced to represent the probability of landslide deformation. The probability is denoted as P1, expressed by the formula:


$$P\text{1}=\frac{\text{1}}{\left(\text{1}+e^{-F(V,P,Pe)}\right)}$$ (15)

$$F(V,P,Pe)=\beta_{\text{0}}+\beta_{\text{1}}V+\beta_{\text{2}}P+\beta_{\text{3}}Pe+\beta_{\text{4}}V^{\text{2}}+\beta_{\text{5}}P^{\text{2}}+\beta_{\text{6}}{Pe}^{\text{2}}+\beta_{\text{7}}VP+\beta_{\text{8}}VPe+\beta_{\text{9}}PPe$$ (16)

Where β_4_, β_5_and β_6_ are coefficients for quadratic terms, and β_7_, β_8_, and β_9_ are coefficients for interaction terms.

Using 24-hour monitoring data as the basis, parameters were fitted through nonlinear least squares, and insignificant terms were removed to simplify the model to:


$$F(V,P,Pe)=\beta_{\text{0}}+\beta_{\text{1}}V+\beta_{\text{2}}P+\beta_{\text{3}}Pe+\beta_{\text{4}}V^{\text{2}}+\beta_{\text{5}}P^{\text{2}}+\beta_{\text{6}}{Pe}^{\text{2}}$$ (17)

Since weakly correlated interaction terms (*VP, VPe, PPe*) were initially excluded in the 24-hour to 6-hour monitoring data, these terms were reintroduced to improve the model’s accuracy for short-period data (such as 1-hour and 3-hour periods). This adjustment aims to better capture the sensitivity of short-period data to landslide deformation, particularly during rapid changes in rainfall and displacement rates. The model parameters for each monitoring period are presented in Table 6.

**Table 6:** Fitting parameters for each monitoring cycle.

Monitoring Period	*β*_*0*_	*β*_*1*_	*β*_*2*_	*β*_*3*_	*β*_*4*_	*β*_*5*_	*β*_*6*_	*β*_*7*_	*β*_*8*_
24h	−8.5	0.15	0.001	0.025	0.0002	0.0001	0.0008	/	/
12h	−9.5	0.12	0.045	0.035	0.0002	0.0035	0.0009	/	/
6h	−8.7	0.16	0.065	0.035	0.00025	0.0050	0.0009	/	/
3h	−5.0	0.25	0.08	0.12	0.0003	0.0039	0.00020	0.005	0.0005
1h	−4.0	0.35	0.09	0.15	0.00035	0.0040	0.00025	0.008	0.0006

**Fig 16:**
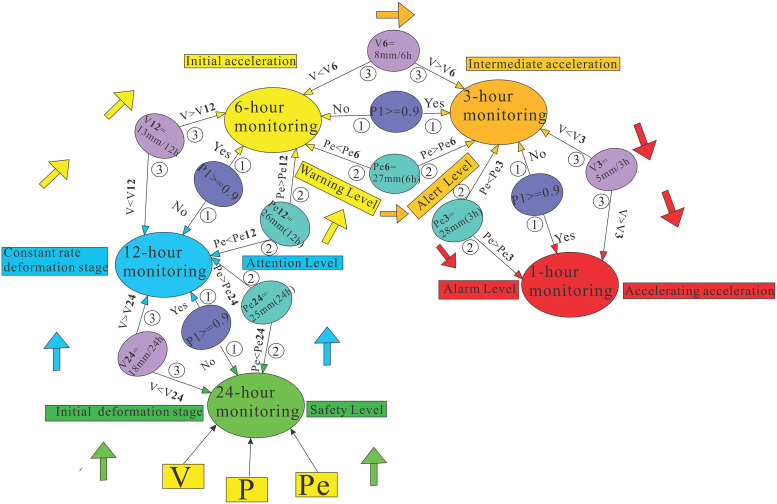
Dynamic early warning model.

Through refined analysis of rainfall and displacement rates in the minimum and maximum step-like intervals of the Tanjiawan landslide, a series of thresholds were determined. Subsequently, by integrating the regression model and the function *F(V, P, Pe)*, a probability-based landslide warning model was developed, providing a structured warning framework for the Tanjiawan landslide. The specific model is shown in Fig 16.

This multi-level dynamic early warning model (Fig 16) is based on the landslide deformation process (including initial deformation, constant velocity deformation, and accelerated deformation stages). Through an adaptive monitoring cycle adjustment mechanism (24h to 1h), it achieves early warning for different deformation stages, corresponding to five warning levels: Safe, Caution, Alert, Warning, and Alarm.

When dynamically adjusting the monitoring cycle, the model adopts a multi-level discrimination system with clear priorities and follows a strict sequential logic for state determination: First, when the combined effect of displacement rate, antecedent rainfall, and current rainfall leads to a landslide probability P1 greater than 0.9 (Condition①), this highest-priority condition immediately triggers intensified monitoring once met. If not satisfied, the system then checks whether the antecedent rainfall has reached the analysis threshold (Condition②). At this point, even if the probability does not reach 0.9, the landslide enters a sensitive stage. If neither of the first two conditions is triggered, it finally examines whether the displacement rate shows anomalies (Condition③). When the displacement rate exceeds the set threshold, the model automatically adjusts the monitoring cycle.

This hierarchical discrimination mechanism ensures deterministic and unique system responses while comprehensively covering various risk scenarios from comprehensive probability-based warnings to single-parameter anomalies. The model comprehensively considers the synergistic effects of displacement rate (V), current rainfall (P), and antecedent rainfall (Pe), with particular emphasis on the critical role of displacement rate and antecedent rainfall accumulation, while avoiding missed reports through special point monitoring. When monitoring parameters exceed safety thresholds, the monitoring cycle is automatically intensified, and the warning level is correspondingly elevated. This priority-based discrimination logic effectively prevents confusion caused by state overlap, significantly enhances the reliability and adaptability of the early warning system, ensures effective tracking of the landslide evolution process at different stages, and provides reliable support for disaster response.

### Comparative analysis with existing rainfall thresholds and model validation

The dynamic early warning model proposed in this study exhibits multidimensional complementarity with traditional rainfall threshold methods in terms of warning mechanisms. Classical rainfall threshold models, such as the intensity-duration model and cumulative rainfall-duration model, establish statistical relationships between rainfall parameters and landslide probability, providing an important theoretical basis for regional landslide early warning [52]. These statistical models based on historical disaster data demonstrate significant advantages in large-scale regional risk assessment, particularly in areas with limited monitoring data, enabling preliminary risk screening at relatively low costs and offering scientific support for regional disaster prevention planning.

The main innovation of our model lies in the deep integration of real-time displacement monitoring data with rainfall parameters. This multi-parameter warning approach aligns with recent developments in slope monitoring [53]. Specifically, the monitoring case from August 28–30, 2020 demonstrates its unique value: while traditional I-D threshold models would indicate low risk due to zero rainfall during the monitoring period, our model successfully identified a significant deformation phase with a peak displacement rate of 39.57 mm/day by capturing the hysteretic deformation response of rock and soil mass triggered by antecedent effective rainfall (19.3–44.8 mm). This comparative result indicates that incorporating displacement monitoring parameters can effectively compensate for the limitations of traditional rainfall thresholds in identifying hysteretic deformation effects, consistent with the multi-source data integration concept proposed by Zhang et al. (2025) [54].

In practical applications, the two methods demonstrate different suitable scenarios. Traditional threshold methods are more appropriate for preliminary risk screening at regional scales, while our model, with its multi-temporal monitoring architecture (24-hour to 1-hour), enables dynamic tracking of deformation processes in key slopes. This refined monitoring capability echoes the “graded warning” system advocated by Piciullo et al. (2018) [55]. It should be specifically noted that our model is not intended to replace traditional threshold methods, but rather to provide more comprehensive decision-making information for risk management of important engineering slopes. In practical applications, a hierarchical warning system could be established: employing traditional rainfall thresholds for preliminary screening at regional scales, followed by focused monitoring of identified high-risk areas using our model, thereby forming a complementary warning network. This combined application mode ensures both the coverage of regional warnings and improves monitoring accuracy in critical areas, aligning with the development direction of modern geohazard warning systems.

## Discussion

The dynamic early warning model constructed in this study, based on monitoring data from the Tanjiawan landslide, is primarily applicable to rainfall-triggered landslides exhibiting typical step-like deformation characteristics. The warning threshold system of the model is established by analyzing the statistical relationships among antecedent effective rainfall, current rainfall, and displacement rates. Its core value lies in establishing a hierarchical warning framework with displacement rate (V), current rainfall (P), and antecedent effective rainfall (Pe) as key parameters. Therefore, although the model is constructed based on a single case, its core driving factors possess universality, and the logical framework of hierarchical warning is transferable—when applied to different landslides, site-specific probability models and threshold parameters can be recalibrated using local monitoring data, while the warning decision-making process remains consistent. Furthermore, by dynamically adjusting the monitoring period (e.g., from 24h to 1h), the model is not only suitable for step-like deformation but also capable of capturing the nonlinear acceleration behaviors common in various landslides as they approach failure, thereby optimizing the monitoring of the “pre-failure acceleration phase.”

However, it must be explicitly stated that this model has significant limitations. First, the model treats reservoir water-level fluctuations as relatively slowly varying boundary conditions, and the current threshold system does not explicitly account for their dynamic effects. This is primarily based on three practical considerations: the significant temporal correlation between deformation and rainfall events during the monitoring period, the lack of complete periodic water-level–displacement response data, and the need to ensure immediate warning capability in rainfall-triggered scenarios. Therefore, the proposed thresholds should be precisely defined as empirical warning thresholds reflecting the rainfall-triggering effects under specific reservoir operation conditions. Second, the model is essentially data-driven and empirical, involving necessary simplifications of complex physical processes. Its warning effectiveness heavily depends on the quality of high-frequency, continuous displacement and rainfall monitoring data. Third, the model’s warning capability may be insufficient in the early stages of deformation or for sudden-onset landslides, and its direct applicability to landslides with different geological conditions and scales still requires systematic validation.

Based on the above limitations, future research should focus on three directions for further development: First, conducting cross-regional, multi-case systematic validation to assess the model’s warning effectiveness and applicability boundaries in different geological environments and deformation characteristics. Second, promoting the integration of multi-source information to establish a comprehensive warning model that incorporates “rainfall–reservoir water level–displacement” and even additional parameters such as groundwater levels and deep displacement, while enhancing mechanistic interpretation through integration with physical models. Third, researching dynamic adaptive updating mechanisms for warning thresholds and developing a prototype system for long-term operational testing. Only through such systematic validation and in-depth research can the methodological approach presented in this case study evolve into a landslide early warning technology with broader applicability and higher reliability.

## Conclusions

This study focuses on the Tanjiawan landslide, introducing the concept of “a single rainfall process” to characterize the rainfall events influencing landslide deformation. Through detailed analysis of deformation characteristics such as displacement and displacement rate under rainfall conditions, the antecedent rainfall threshold (*Pe*), current rainfall (*P*), and displacement rate threshold (*V*) were determined. Finally, based on the deformation features of the Tanjiawan landslide, a refined dynamic early warning model was developed by integrating the function *F*(*V*, *P*, *Pe*) with a logistic regression model.The main conclusions are summarized as follows:

(1) The deformation of the Tanjiawan landslide is closely related to rainfall processes, with the cumulative displacement curve exhibiting distinct “step-like” characteristics and the displacement rate showing a “lag-attenuation” phenomenon. The displacement rate peaks 1–2 days after rainfall ceases, then gradually decreases until returning to normal levels, with this process lasting approximately 10 days.
(2) Through refined analysis of rainfall and displacement rates under different monitoring cycles, it was found that finer monitoring intervals enable more precise capture of landslide deformation dynamics, yielding more reliable thresholds for displacement rates and antecedent rainfall. Particularly under external influences such as rainfall, short-interval monitoring (6h, 3h, and 1h) can better detect subtle deformation fluctuations, thereby providing more robust criteria for landslide early warning and prediction.
(3) Based on the displacement rate (*V*), current rainfall (*P*), and effective prior rainfall (*Pe*) obtained from refined analysis across different monitoring periods, combined with the function *F(V, P, Pe)* and a logistic regression model, a landslide warning model with dynamic adjustment capabilities was developed. This model emphasizes the critical roles of displacement rate thresholds and effective prior rainfall thresholds under different monitoring periods, dynamically adjusting the monitoring period (from 24h to 1h) to facilitate early warning and prediction.

## Supporting information

S1 File Supporting data tables. Contains all data tables referenced in the manuscript.
(DOCX)
