# Supplementary material for: Research on a dynamic early warning model based on refined threshold analysis: Case study of the Tanjiawan Landslide
Source: PLoS One. 2026 Feb 9;21(2):e0339689. doi: 10.1371/journal.pone.0339689 (PMC12885304; doi:10.1371/journal.pone.0339689)
Supplement: S1 File — Contains all data tables referenced in the manuscript. (DOCX) [file pone.0339689.s001.docx]

Table 1

Rainfall Parameters

| Time Period | Average Daily Rainfall （mm） | Number of Days with Daily Rainfall >10 mm | Total Rainfall (mm) |
| --- | --- | --- | --- |
| 2020/06/01-2020/12/31 | 3.45 | 25 | 739.4 |
| 2021/01/01-2021/07/01 | 1.83 | 8 | 334.2 |

Table 2

Information Sheet of Seven Step Deformations

| Step-like Deformation (n) | Step Interval | Displacement Increment (mm) | Average Displacement Rate (mm/d) |
| --- | --- | --- | --- |
| 1 | June 22, 2020 - July 11, 2020 | 1505.05 | 75.25 |
| 2 | July 18, 2020 - August 3, 2020 | 819.93 | 48.16 |
| 3 | August 28, 2020 - September 4, 2020 | 140.14 | 18.84 |
| 4 | October 4, 2020 - October 13, 2020 | 1569.62 | 173.92 |
| 5 | November 24, 2020 - December 4, 2020 | 181.46 | 16.50 |
| 6 | July 9, 2021 - July 22, 2021 | 287.0 | 20.5 |
| 7 | August 22, 2021 - September 1, 2021 | 1788.70 | 162.54 |

Table 3.

Rainfall and displacement rates under different monitoring periods (step deformation with minimal displacement increment)

| Monitoring Period (T_24/12/6/3/1_) | Rainfall Date (YYYY/MM/DD) | Current Rainfall (mm) | Effective Prior Rainfall (mm) | Displacement Rate (mm/24h,12h,6h,3h,1h) |
| --- | --- | --- | --- | --- |
| T_24_=[0，8] | 2020/8/28 | 0 | 31.6 | 10.58 |
|  | 2020/8/29 | 0 | 25.0 | 39.57 |
|  | 2020/8/30 | 0 | 19.3 | 24.92 |
| T_12_=[0，15] | 2020/08/28 00:00-12:00 | 0 | 44.8 | 5.9 |
|  | 2020/08/28 12:00-24:00 | 0 | 39.1 | 7.68 |
|  | 2020/08/29 00:00-12:00 | 0 | 36.7 | 22.65 |
|  | 2020/08/29 12:00-24:00 | 0 | 31.6 | 20.49 |
|  | 2020/08/30 00:00-12:00 | 0 | 29.5 | 16.51 |
|  | 2020/08/30 12:00-24:00 | 0 | 23.0 | 6.91 |
| T_6_=[0，31] | 2020/08/28 12:00-18:00 | 0 | 31.6 | 7.71 |
|  | 2020/08/28 18:00-24:00 | 0 | 29.5 | 10.8 |
|  | 2020/08/29 00:00-06:00 | 0 | 29.5 | 12.48 |
|  | 2020/08/29 06:00-12:00 | 0 | 28.5 | 14.81 |
|  | 2020/08/29 18:00-24:00 | 0 | 27.2 | 12.82 |
|  | 2020/08/30 00:00-06:00 | 0 | 26.1 | 11.83 |
| T_3_=[0，63] | 2020/08/28 18:00-21:00 | 0 | 32.4 | 4.96 |
|  | 2020/08/28 21:00-24:00 | 0 | 31.6 | 5.01 |
|  | 2020/08/29 00:00-03:00 | 0 | 30.3 | 5.27 |
|  | 2020/08/29 03:00-06:00 | 0 | 29.8 | 5.21 |
|  | 2020/08/29 06:00-09:00 | 0 | 29.5 | 5.36 |
|  | 2020/08/29 09:00-12:00 | 0 | 29.1 | 5.64 |
|  | 2020/08/29 12:00-15:00 | 0 | 28.2 | 5.14 |
|  | 2020/08/29 15:00-18:00 | 0 | 27.5 | 4.68 |
|  | 2020/08/29 18:00-21:00 | 0 | 26.7 | 4.42 |
|  | 2020/08/29 21:00-24:00 | 0 | 26.0 | 3.41 |
|  | 2020/08/30 00:00-03:00 | 0 | 26.5 | 4.55 |
|  | 2020/08/30 03:00-06:00 | 0 | 25.2 | 3.51 |
| T_1_=[0，127] | 2020/08/28 22:00-23:00 | 0 | 31.6 | 2.3 |
|  | 2020/08/28 23:00-24:00 | 0 | 31.6 | 2.5 |
|  | 2020/08/29 00:00-01:00 | 0 | 31.6 | 2.3 |
|  | 2020/08/29 01:00-02:00 | 0 | 31.3 | 2.9 |
|  | 2020/08/29 02:00-03:00 | 0 | 30.3 | 3 |
|  | 2020/08/29 03:00-04:00 | 0 | 29.6 | 3.1 |
|  | 2020/08/29 12:00-13:00 | 0 | 29.2 | 3.7 |
|  | 2020/08/29 13:00-14:00 | 0 | 29.2 | 4.1 |
|  | 2020/08/29 14:00-15:00 | 0 | 29.2 | 4.1 |
|  | 2020/08/29 15:00-16:00 | 0 | 29.2 | 3.9 |
|  | 2020/08/29 21:00-22:00 | 0 | 28.4 | 1 |
|  | 2020/08/29 22:00-23:00 | 0 | 28.0 | 1.3 |
|  | 2020/08/29 23:00-24:00 | 0 | 28.0 | 1.1 |
|  | 2020/08/30 00:00-01:00 | 0 | 27.9 | 1.3 |
|  | 2020/08/30 01:00-02:00 | 0 | 27.7 | 1.1 |

Table 4.

Rainfall and displacement rates under different monitoring periods (step deformation with maximum displacement increment)

| Monitoring Period (T_24/12/6/3/1_) | Rainfall Date (YYYY/MM/DD) | Current Rainfall (mm) | Effective Prior Rainfall (mm) | Displacement Rate (mm/24h,12h,6h,3h,1h) |
| --- | --- | --- | --- | --- |
| T_24_=[0，11] | 2021/8/27 | 17.6 | 82.6 | 403.62 |
|  | 2021/8/28 | 0.6 | 70.7 | 800.81 |
|  | 2021/8/29 | 0 | 71.3 | 234.00 |
| T_12_=[0，21] | 2021/08/27 00:00-12:00 | 17.4 | 74.6 | 134.05 |
|  | 2021/08/27 12:00-24:00 | 0.2 | 88.1 | 269.00 |
|  | 2021/08/28 00:00-12:00 | 0.6 | 86.6 | 479.27 |
|  | 2021/08/28 12:00-24:00 | 0.0 | 83.2 | 321.53 |
|  | 2021/08/29 00:00-12:00 | 0.0 | 77.3 | 169.44 |
|  | 2021/08/29 12:00-24:00 | 0.0 | 71.3 | 65.12 |
| T_6_=[0，43] | 2021/08/27 12:00-18:00 | 0.20 | 88.1 | 106.93 |
|  | 2021/08/27 18:00-24:00 | 0.00 | 87.5 | 178.42 |
|  | 2021/08/28 00:00-06:00 | 0.6 | 86.6 | 232.09 |
|  | 2021/08/28 06:00-12:00 | 0 | 85.4 | 247.19 |
|  | 2021/08/28 18:00-24:00 | 0 | 79.5 | 101.03 |
|  | 2021/08/29 00:00-06:00 | 0 | 77.3 | 96.29 |
| T_3_=[0，87] | 2021/08/27 18:00-21:00 | 0 | 88.0 | 80.96 |
|  | 2021/08/27 21:00-24:00 | 0 | 88.3 | 97.46 |
|  | 2021/08/28 00:00-03:00 | 0 | 83.7 | 122.18 |
|  | 2021/08/28 03:00-06:00 | 0 | 81.4 | 124.34 |
|  | 2021/08/28 06:00-09:00 | 0 | 84.3 | 122.85 |
|  | 2021/08/28 09:00-12:00 | 0 | 85.0 | 114.45 |
|  | 2021/08/28 12:00-15:00 | 0 | 87.0 | 106.06 |
|  | 2021/08/28 15:00-18:00 | 0 | 85.6 | 60.25 |
|  | 2021/08/28 18:00-21:00 | 0 | 85.4 | 40.78 |
|  | 2021/08/28 21:00-24:00 | 0 | 84.0 | 122.18 |
|  | 2021/08/29 00:00-03:00 | 0 | 83.2 | 49.33 |
|  | 2021/08/29 03:00-06:00 | 0 | 81.4 | 46.95 |
| T_1_=[0，263] | 2021/08/27 22:00-23:00 | 0 | 88.3 | 32.4 |
|  | 2021/08/27 23:00-24:00 | 0 | 88.3 | 34.7 |
|  | 2021/08/28 00:00-01:00 | 0 | 88.3 | 35.7 |
|  | 2021/08/28 01:00-02:00 | 0 | 88.2 | 37.2 |
|  | 2021/08/28 02:00-03:00 | 0 | 88.0 | 37.0 |
|  | 2021/08/28 03:00-04:00 | 0 | 87.9 | 39.7 |
|  | 2021/08/28 12:00-13:00 | 0 | 85.4 | 33.6 |
|  | 2021/08/28 13:00-14:00 | 0 | 85.3 | 31.7 |
|  | 2021/08/28 14:00-15:00 | 0 | 85.3 | 30.8 |
|  | 2021/08/28 15:00-16:00 | 0 | 84.0 | 28.3 |
|  | 2021/08/28 21:00-22:00 | 0 | 83.2 | 22.4 |
|  | 2021/08/28 22:00-23:00 | 0 | 83.2 | 19.8 |
|  | 2021/08/28 23:00-24:00 | 0 | 83.2 | 20.9 |
|  | 2021/08/29 00:00-01:00 | 0 | 83.2 | 15.4 |
|  | 2021/08/29 01:00-02:00 | 0 | 83.1 | 15.6 |

Table 5

Threshold Values under Different Monitoring Periods

| Monitoring Period | Effective Prior Rainfall Threshold | Displacement Rate Threshold |
| --- | --- | --- |
| 24h | 25mm | 18mm/24h |
| 12h | 26mm | 13mm/12h |
| 6h | 27mm | 8mm/6h |
| 3h | 28mm | 5 mm/3h |
| 1h | 29mm | 2mm/1h |

Table 6

Fitting Parameters for Each Monitoring Cycle

| Monitoring Period | *β_0_* | *β_1_* | *β_2_* | *β_3_* | *β_4_* | *β_5_* | *β_6_* | *β_7_* | *β_8_* |
| --- | --- | --- | --- | --- | --- | --- | --- | --- | --- |
| 24h | -8.5 | 0.15 | 0.001 | 0.025 | 0.0002 | 0.0001 | 0.0008 | / | / |
| 12h | −9.5 | 0.12 | 0.045 | 0.035 | 0.0002 | 0.0035 | 0.0009 | / | / |
| 6h | −8.7 | 0.16 | 0.065 | 0.035 | 0.00025 | 0.0050 | 0.0009 | / | / |
| 3h | −5.0 | 0.25 | 0.08 | 0.12 | 0.0003 | 0.0039 | 0.00020 | 0.005 | 0.0005 |
| 1h | −4.0 | 0.35 | 0.09 | 0.15 | 0.00035 | 0.0040 | 0.00025 | 0.008 | 0.0006 |
